# Isoeugenol and Hybrid Acetamides against *Candida albicans* Isolated from the Oral Cavity

**DOI:** 10.3390/ph13100291

**Published:** 2020-10-03

**Authors:** Daianne Medeiros, José Oliveira-Júnior, Jefferson Nóbrega, Laísa Cordeiro, Jeane Jardim, Helivaldo Souza, Gracielle Silva, Petrônio Athayde-Filho, José Barbosa-Filho, Luciana Scotti, Edeltrudes Lima

**Affiliations:** 1 Department of Pharmaceutical Science, Health Sciences Center, Federal University of Paraíba, 58033-455 João Pessoa, Paraíba, Brazil; daiannemedeiros1@gmail.com (D.M.); joseklidemberg@gmail.com (J.O.-J.); jeffersonrodriguesn@hotmail.com (J.N.); laisavilar@gmail.com (L.C.); jbarbosa@ltf.ufpb.br (J.B.-F.); luciana.scotti@gmail.com (L.S.); edelolima@yahoo.com.br (E.L.); 2 Chemistry Department, Exact and Natural Sciences Center, Federal University of Paraíba, 58033-455 João Pessoa, Brazil; jeanegalindo@hotmail.com (J.J.); gracielletavares@ltf.ufpb.br (G.S.); athayde-filho@quimica.ufpb.br (P.A.-F.)

**Keywords:** *Candida albicans*, isoeugenol, checkerboard method, molecular docking, antifungal activity, oral candidiasis

## Abstract

Isougenol is a phytoconstituent found in several essential oils. Since many natural products are potent antimicrobials, the synthesis of hybrid molecules—combining the chemical skeleton of the phytochemical with synthetic groups—can generate substances with enhanced biological activity. Based on this, the objective of this study was to evaluate the antifungal activity of isoeugenol and hybrid acetamides against *Candida albicans* isolated from the oral cavity. The methodologies used were the determination of minimum inhibitory concentration (MIC), minimum fungicidal concentration (MFC), action on fungal micromorphology, interaction test with nystatin by the checkerboard method and molecular docking study with important enzymes in the maintenance of fungal viability. The synthetic molecules did not demonstrate significant antifungal activity in vitro. The isoeugenol MIC and MFC varied between 128 and 256 µg/mL, being the phytoconstituent able to interfere in the formation of blastoconid and chlamydoconid structures, important in the pathogenic process of the species. The molecular docking study revealed that isoeugenol is a potential inhibitor of the enzymes 14-α-demethylase and delta-14-sterol reductase, interfering in the fungal cell membrane biosynthesis. Thus, this research provides clearer expectations for future pharmacological studies with isoeugenol and derived molecules, aiming at its therapeutic application against infections caused by *Candida* spp.

## 1. Introduction

Candidiasis is an infectious process of clinical importance caused by dimorphic, commensal, opportunistic fungi, belonging to the genus *Candida* [1]. It is an infection of considerable public relevance, since yeasts of this genus are responsible for about 80% of hospital fungal infections [2], representing the fourth most common nosocomial pathogen in systemic infections [3], with morbidity and mortality rates between 30–50% [4].

The genus *Candida* comprises of about 150–200 described species, however only fifteen can be pathogenic to humans, with *C. albicans*, *C. glabrata*, *C. parapsilosis*, *C. tropicalis* and *C. krusei* being the most prevalent in different types of candidiasis [5,6,7]. Several virulence factors are directly related to the capacity of these species to infect their hosts, including: the ability to morphological transition between the yeast and hyphal states, phenotypic plasticity, biofilm formation, production and secretion of hydrolytic enzymes such as phosphatases, lipases and hemolysins, adhesin and invasin expression and growth via tigmotropism [8,9].

Among fungal infections of mucous membranes, oral candidiasis is one of the most prevalent, with clinical features presenting as the appearance of whitish-yellow plaques or reddish nodules, of various aspects, found adhered to the oral mucosa, tongue, palate and gums [10]. Several predisposing factors are associated with a high prevalence of oral candidiasis, including nighttime use of dental prostheses, decreased salivary pH, dry mouth, prolonged use of antibiotic therapy and immunosuppressive drugs, nutritional deficiency, endocrine disorders and increased life expectancy of the population [6,11,12].

The treatment of oral candidiasis is based on the use of antifungal agents, especially nystatin [1,13]. However, with the increase in fungal resistance to antimicrobial agents and the limited number of antifungal molecules available today, the search for new bioactive compounds capable of inhibiting *Candida* growth and demonstrating low toxicity to human cells is increasing [14,15].

In this context, medicinal plants represent a vast source of new pharmacologically active molecules for the treatment of fungal diseases [15,16,17]. Among the compounds isolated from plants, phenylpropanoids constitute a group of phytoconstituents derived from phenylalanine widely found in essential oils such as *Syzygium aromaticum* (clove), *Myristica frangrans* (walnut shown), *Ocimum basilicum* (basil) and *Laurus nobilis* (louro) oils [15,18,19,20], with isoeugenol being a representative of this class of molecules [21,22].

Numerous publications report antimicrobial, antioxidant, anti-inflammatory, anti-tumor and analgesic properties of phenylpropanoids [15,19,23,24]. However, there are still few published studies on the antifungal activity of isoeugenol on *Candida* spp., demonstrating the need for further studies to understand this property on strains of this genus.

On the other hand, the amide group is widely found and is of fundamental importance for the biological and material properties of a large number of compounds created by man and present in nature [25]. There are some reports of acetamide derivatives as antibacterial and antifungal agents [26,27,28]. As Aschale [29] explains, there was considerable interest in making substitutions for acetanilide derivatives because this group is present in several synthetic drugs related to a wide range of biological activities, such as antibacterial, antiviral, antifungal and anthelmintic.

Due to the potent antimicrobial activity of natural products, these substances can serve as targets for molecular changes that aim to improve their biological activity and, among these modifications, combinatorial syntheses that produce hybrid molecules allow the joining of phytoconstituents with other chemical classes, obtaining interesting pharmacological results [30].

Based on this, the objective of this study was to evaluate the antifungal activity of isoeugenol and hybrid acetamides against *Candida albicans* isolated from the oral cavity, investigating their effects through in vitro and in silico analyzes.

## 2. Results

### 2.1. Minimum Inhibitory Concentration (MIC) and Minimum Fungicidal Concentration (MFC) Determination

The screening of hybrid acetamides and isoeugenol antifungal activities on *C. albicans* revealed that the synthetic molecules did not show a significant capacity to inhibit fungal growth (Table 1), in contrast to isoeugenol, which presented MIC ranging from 128 to 256 µg/mL.

Among the analyzed molecules, isoeugenol showed superior antifungal activity and, for this reason, the following tests were carried out with this phytoconstituent.

The MIC and MFC values of isoeugenol and nystatin against *C. albicans* are shown in Table 2. The isoeugenol MIC and MFC values ranged between 128 µg/mL and 256 µg/mL. For nystatin, the MIC values were 8 µg/mL, while the MFC varied between 8 µg/mL and 32 µg/mL. The control of strains viability showed fungal growth in all wells and the sterility of the medium did not have any fungal growth.

### 2.2. Interference of Isoeugenol on C. albicans Micromorphology

**Figure 1:**
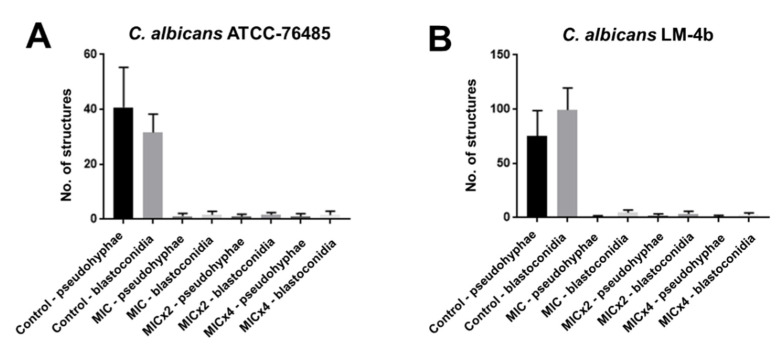
Growth of pseudo-hyphae and blastoconidia of (**A**) American Type Culture Collection (ATCC)-76485 and (**B**) LM-4b in groups treated (MIC, MIC × 2, MIC × 4) and not treated (negative control) with isoeugenol.

The test of possible interferences of isoeugenol on the micromorphology of *C. albicans* American Type Culture Collection (ATCC)-13803 and LM-4b was carried out with the objective of verifying the growth or absence of characteristic structures, blastoconidia, chlamydoconidia and pseudo-hyphae, at the concentrations of MIC, MIC × 2, MIC × 4 and in absence of isoeugenol. The number of virulence structures, blastoconidia and pseudo-hyphae, was performed by averaging the structure counts per field, in five fields analyzed from each slide (Figure 1).

**Figure 2:**
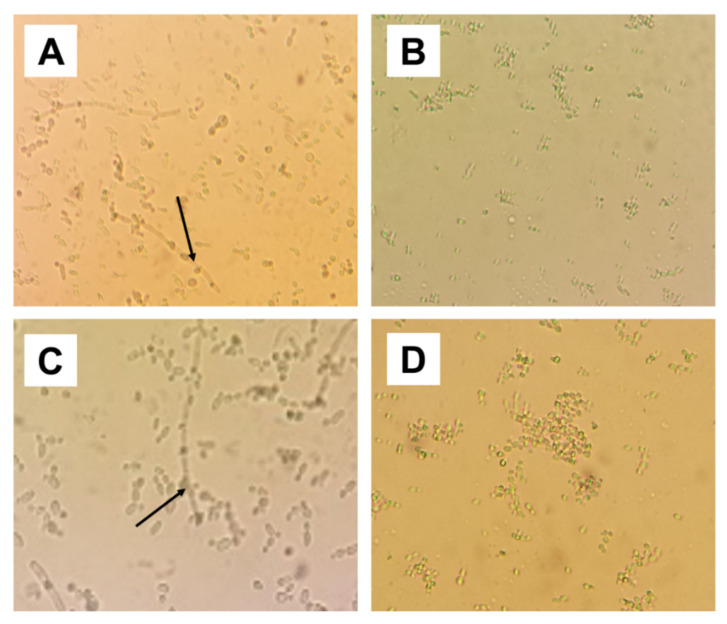
Micromorphology analysis. (**A**) *C. albicans* ATCC 76485 in the absence of isoeugenol (negative control), showing virulence structures of pseudo-hyphae and blastoconidia. (**B**) *C. albicans* ATCC 76485 treated with isoeugenol, there is a lack of pseudo-hyphae and a reduced number of blastoconidia. (**C**) *C. albicans* LM-4b in the absence of isoeugenol (negative control), showing a large amount of blastoconidia and pseudo-hyphae. (**D**) *C. albicans* LM-4b under the effect of isoeugenol, absence of blastoconidia. Black arrow: Chlamydocide resistance structures.

Chlamydoconidia resistance structures were not found in the MIC, MIC × 2 and MIC × 4 concentrations of isoeugenol (Figure 2).

### 2.3. In Vitro Association Assay

The isoeugenol and nystatin interaction was evaluated according to the checkerboard methodology, which is an in vitro laboratory technique that allows the interaction of two or more molecules through the construction of a two-dimensional matrix between the tested substances [31]. The data obtained in this study demonstrated that the interaction of isoeugenol with nystatin was indifferent, with index values fractional inhibitory concentration (FIC) = 3.09 and FIC = 2.10, for *C. albicans* LM-4b and *C. albicans* ATCC-76485, respectively (Table 3).

### 2.4. Molecular Docking Analysis

The molecular docking study revealed that the isoeugenol fitted into the active site of 14-α-demethylase enzymes (Protein Data Bank (PDB) id: 5FSA) [32], delta-14-sterol reductase (PDB id: 4QUV) [33], lanosterol 14-α-demethylase (PDB id: 4LXJ) [34] and 1,3-β-glucan synthase (PDB id: 2J0Y) [35] responsible for the biosynthesis and maintenance of the plasma membrane fungal and cell wall formation. The binding energies between the molecules and the target protein (MolDock score) are shown in Table 4.

**Figure 3:**
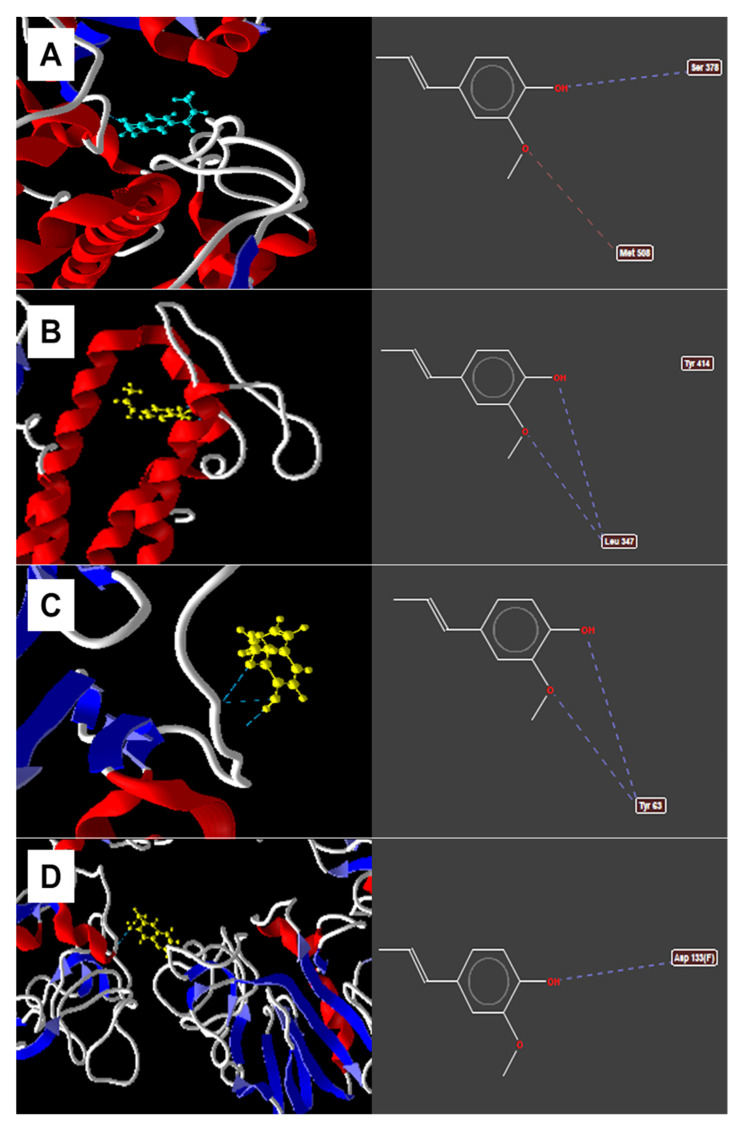
Observed interactions between isoeugenol and (**A**) 14-α-demethylase, (**B**) delta-14-α-reductase, (**C**) lanosterol-14-α-demethylase, (**D**) 1,3-β-glucan synthase. Dashed blue lines indicate hydrogen bonds. Dashed red lines indicate electrostatic connections.

Figure 3 shows the results of the study of isoeugenol docking with the evaluated enzymes. Delta-14-sterol reductase was the enzyme with the best energy value coupled with isoeugenol (−84.5773 kcal/mol), showing hydrogen bonds of the methoxy group and free hydroxyl with Leu347 and steric bonding with the Tyr414 residue. After delta-14-sterol reductase, 14-α-demethylase was the second enzyme to demonstrate the best energy value (−69.4023 kcal/mol) with hydrogen bonds of hydroxyl oxygen with Ser378 residue and electrostatic oxygen from the methoxy group with the Met508 residue.

## 3. Discussion

Although the combinatorial synthesis of natural products with synthetic molecules may result in an improvement in biological activity [30], in this study, the hybrid acetamides obtained from isoeugenol did not show considerable antifungal activity, as the original phytoconstituent was more potent against *C. albicans* than the synthetic molecules tested.

The results obtained indicated that isoeugenol has remarkable antifungal activity. The data demonstrate that the lowest concentration of isoeugenol capable of inhibiting the growth of *C. albicans* was 128 µg/mL. Similar results were found by Bhatia et al. [36] who reported that the MIC value of isoeugenol was between 100–250 µg/mL for *Candida* ssp. resistant and sensitive to fluconazole, and by Zemek et al. [37] who determined that the MIC value = 100 µg/mL of isoeugenol for *C. albicans*. In contrast, Gallucci et al. [23] determined that the MIC value of isoeugenol capable of inhibiting the growth of fluconazole-resistant *C. albicans* was 0.17 mg/mL. Other studies have also revealed the ability of isoeugenol to inhibit the growth of pathogenic Gram-positive, Gram-negative and *Mycobacterium fortuitum* bacteria [38], in addition to fungi of the genus *Aspergillus*, *Saccaromyces* spp. and *Fusarium parasiticus* [37,38,39].

The MFC of isoeugenol was identical in all strains analyzed, whereas for nystatin the value of MFC was 3× the value of MIC against *C. albicans* 11b, *C. albicans* 13b and *C. albicans* ATCC 76485. According to Siddique et al. [40] a compound has a fungistatic action when the MFC/MIC ratio is ≥4 and fungicidal when MFC/MIC < 4. Thus, isoeugenol showed fungicidal action in all strains evaluated, while nystatin in only 50% of the strains analyzed, as shown in Table 1. The results found by Peixoto et al. [19] corroborate those observed in this study, in which the value of MFC/MIC of the *Laurus nobilis* essential oil against *C. albicans* coming from the oral cavity was <4. The authors attributed the anti-*Candida* activity of *L. nobilis* to isoeugenol, a major compound (53.5%) found in essential oil [19].

The antimicrobial activity of isoeugenol can be attributed to the structural and molecular parameters of the phytoconstituent, in addition to the variation according to the species analyzed and the number of substituents on the molecule’s aromatic ring [37,41]. The presence of free hydroxyl (-OH) from the aromatic chain of isoeugenol is one of the structural parameters involved in the antimicrobial activity of this phytoconstituent, however, molecular properties such as hydrophobicity, refractivity and molecular geometry also seem to be related to the inhibitory capacity of isoeugenol on *Candida* spp. [37,40,41].

The ability of *C. albicans* to present structures such as blastoconidia and pseudo-hyphae characterizes an important virulence factor of the genus in the infectious process, since the presence of these structures hinders phagocytosis of the pathogen by the cells of the host immune system [8]. Isougenol has been shown to be able to reduce the formation of virulence structures (chlamidoconidia, blastoconidia and pseudo-hyphae) of *C. albicans*, suggesting that it is an effective product in controlling the establishment and progression of candidiasis.

Although the mechanisms of action of isoeugenol are not fully understood, the literature reports that the phytoconstituent is capable of inhibiting the growth of *Candida* spp. because it affects the formation of the fungal cell wall and destabilizes the plasma membrane through its interaction with proton pumps, consequently causing destruction of the microorganism, considering that this enzyme regulates important physiological processes for the viability of fungal cells [19,36]

The association study of isoeugenol with nystatin on *C. albicans* showed indifference, that is, when combined, the two compounds did not interact with each other to act negatively or positively on *C. albicans*. Although the absence of interaction between the phytoconstituent isoeugenol and the standard drug nystatin has been observed, the results of this study do not preclude further research that seeks the interaction profile of isoeugenol with other licensed antifungal drugs or natural molecules.

Abourashed et al. [42] analyzed the combinatory effect of isoeugenol with the natural molecules 6-paradol and 6-shogaol against *C. albicans* and isoeugenol with isoniazid antibiotic against *Mycobacterium smegmatis* and as a result, obtained strong synergism between the compounds evaluated on *C. albicans* and *M. smegmatis*. Thus, further studies should be carried out to evaluate the association profile of isoeugenol with antimicrobials of different classes and its potentiating effect with other natural molecules with antimicrobial action.

Studies to elucidate biological activities of different molecules, using in vitro and in silico methods, can be carried out concurrently with the objective of illustrating the possible interactions between test molecules and the active site of the target microorganisms [15].

The evaluation of the possible mechanisms of action of isoeugenol, by the in silico method of molecular docking, showed that it possibly acts in the biosynthesis of the *Candida* cell membrane, since the best values of binding energy were demonstrated in the coupling with delta-enzymes—14-α-reductase and 14-α-demethylase—responsible for the formation of the main sterol present in the fungal plasma membrane: ergosterol [32,33].

The literature does not report any other study that shows interactions between isoeugenol and the evaluated enzymes, confirming the need for further studies aimed at elucidating the antifungal potential of isoeugenol on the biosynthesis of structures essential to fungal cell viability.

## 4. Materials and Methods

### 4.1. Substances

**Figure 4:**
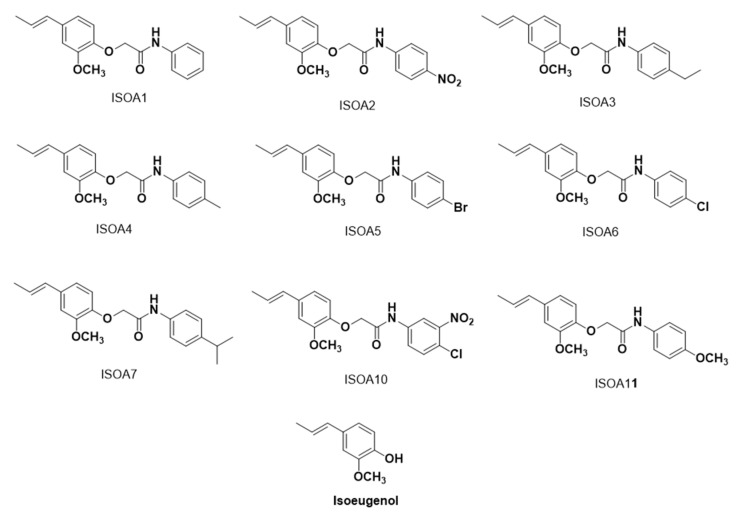
Chemical structures of hybrid acetamides derived from isoeugenol (ISO A1–ISO A11) and isoeugenol.

Isoeugenol and nystatin were purchased from Merck/Sigma-Aldrich^®^ (Darmstadt, Germany). Hybrid acetamides derived from isoeugenol (Figure 4) were synthesized at the Bioenergy and Organic Synthesis Research Laboratory of the Federal University of Paraíba (Brazil). The synthetic routes and the respective characterizations of the substances are shown in the Supplementary Materials. The substances were solubilized in dimethylsulfoxide (DMSO) at 5% and Tween 80 at 2% to obtain emulsions in the necessary concentrations to be used in each in vitro assay of antifungal activity.

### 4.2. Microorganisms

The clinical isolates used in this study belong to the MICOTECA collection of Research Laboratory on Antibacterial and Antifungal Activity of Natural and Synthetic Bioactive Products of the Federal University of Paraíba (Brazil). Five *C. albicans* strains isolated from the oral cavity were used: four clinical isolates (LM-4b, LM-5b, LM-10b, LM-11b, LM-13b) and a standard strain ATCC-76485. All strains were preserved on Sabouraud dextrose agar (SDA) (Difco^®^, Sparks, MD, USA) at 4 °C.

### 4.3. Minimum Inhibitory Concentration (MIC) and Minimum Fungicidal Concentration (MFC) Determination

The microbiological screening and minimum inhibitory concentration (MIC) of the evaluated products were determined by the broth microdilution method using 96-well microdilution plates. Initially, 100 µL of double concentrated Roswell Park Memorial Institute (RPMI)-1640 broth (Merck, Sigma-Aldrich^®^, Darmstadt, Germany) was added to the wells of the plate. Then, 100 µL of the emulsion of the test product were deposited on the first line of the plate and serial dilutions were performed, transferring a 100 µL aliquot from the well containing the most concentrated medium to the subsequent (less concentrated) one, obtaining different concentrations. Finally, 10 µL of the inoculum corresponding to each yeast was added to the wells.

Each inoculum was prepared in sterile 0.9% saline and vortexed for 15 s. Cell density was compared and standardized, using the 0.5 standard of the McFarland scale, which corresponds to 10^6^ colony forming units per millimeter (CFU/mL). Inoculation at 10^5^ CFU/mL was performed by 1:10 dilution and the final inoculum concentration (1–5 × 10^5^ CFU/mL) was confirmed in a Neubauer chamber [43,44,45]. Controls of sterility of the medium, viability of the strains and susceptibility to standard antifungal nystatin 100 international units (IU) were performed in parallel.

The assay was performed in triplicate and the microdilution plates were incubated at 35 °C (±2 °C) for 24 to 48 h for further analysis of fungal growth. MIC is considered the lowest concentration of substance capable of inhibiting the growth of fungi compared to negative control. To interpret the antimicrobial activity of the test products, the following standard values for MIC were determined: <100 µg/mL (good/strong activity); 100–500 µg/mL (moderate activity); 500–1000 µg/mL (weak activity) and MIC > 1000 µg/mL (inactivity) [46].

After determining the MIC, 10 µL aliquots of the supernatant from the wells equivalent to MIC, MIC × 2 and MIC × 4 were transferred to new 96-well plates containing only RPMI-1640 broth and incubated at 35 °C (±2 °C) for 24–48 h. Then the fungal growth was read. MFC is determined as the lowest concentration capable of inhibiting fungal growth. The tests were performed in triplicate. The MFC/MIC ratio was calculated to determine the fungicidal (MFC/MIC < 4) or fungistatic (MFC/MIC ≥ 4) action of the products [31,40].

### 4.4. Effect on the Micromorphology of C. albicans

Strains ATCC-76485 and LM-4b were used to evaluate possible changes in the micromorphology of *C. albicans* cells exposed to isoeugenol. In this assay, microcultures were performed in a humid chamber using solid agar-cornmeal-Tween 80 (Difco^®^, Sparks, MD, USA) [16]. Aliquots of 3 mL of liquid agar-cornmeal-Tween 80, homogenized with MIC, MIC × 2 and MIC × 4 of the test products, were poured into a glass slide and *C. albicans* were seeded and incubated at 35 °C ± 2 °C for 24–48 h.

The slides were analyzed under optical microscopy, at 400× magnification, to observe the formation or absence of characteristic structures such as chlamydoconidia, blastoconidia and pseudo-hyphae. Six slides were prepared and five fields were counted on each slide. The number of virulence structures was calculated using the total average of the structure counts per field and photo documentation was also carried out.

### 4.5. In Vitro Association Study

The effect of the isoeugenol association with nystatin was verified on the *C. albicans* strains ATCC-76485 and LM-4b, determined by microdilution in broth, using the checkerboard technique. To perform this test, initially 100 µL of Saboraud dextrose broth was added to the wells of the 96-well round bottom plate. Then, 50 µL of isoeugenol at MIC/8, MIC/4, MIC/2, MIC, MIC × 2, MIC × 4 and MIC × 8 concentrations were added vertically and 50 µL of nystatin, at these same concentrations, were added horizontally. Finally, the fungal inoculum of *C. albicans* LM-4b and ATCC 76485 was added to the wells and the covered plates were incubated at 35 °C ± 2 °C for 72 h [31,47]. Controls of sterility of the medium and viability of the strains were performed. The assay was performed in triplicate and the fractional inhibitory concentration index (FICI) was determined.

The FICI index was calculated using the sum of FIC_A_ + FIC_B_, where A is isoeugenol and B is nystatin. The FIC_A_, in turn, was determined by the division of the combined MIC_A_/MIC_A_ alone and the FIC_B_ determined by the division of the combined MIC_B_/MIC_B_ alone. The results were interpreted as follows: synergism (FICI ≤ 0.5), indifference (0.5 ≥ FICI < 4) and antagonism (FICI ≥ 4) [31,47].

### 4.6. Molecular Docking Analysis

The three-dimensional structure of isoeugenol was designed using MarvinSkentch 18.5 software and their energy was minimized in the Spartan 18 software. The crystallized structure of the sterol 14-α-demethylase, delta-14-sterol reductase, lanosterol 14-α-demethylase and 1,3-β-glucan synthase were downloaded from the Protein Data Bank (PDB) (www.rcsb.org), under PDB IDs 5FSA [32], 4QUV [33], 4LXJ [34] and 2J0Y [35], respectively. Molecular anchors were determined using Molegro Virtual Docker (MVD) software, (v 6.0.1, Molegro ApS, Aarhus, Denmark), in which the ligand and receptors were subjected to molecular anchorage under GRID 15 Å in radius and 0.30 Å resolution to the enzyme binding site [48].

## 5. Conclusions

It is concluded that the nine hybrid acetamides obtained from isoeugenol tested in this study do not show anti-*Candida albicans* activity. However, other structural modifications can be performed, using isoeugenol as a base, to obtain improvements in antifungal activity. The isoeugenol is an effective antifungal agent against *C. albicans*, as it is able to interfere in the formation of blastoconid and chlamydoconid structures, which is important in the pathogenic process of the species. The molecular docking study revealed that isoeugenol is a potential inhibitor of the enzymes 14-α-demethylase and delta-14-sterol reductase, interfering in the fungal cell membrane biosynthesis. Thus, this research provides clearer expectations for future in vitro and in vivo pharmacological and toxicological studies with isoeugenol and derived molecules, aiming at its therapeutic application against infections caused by *Candida* spp.

## Figures and Tables

**Table 1:** Screening of hybrid acetamides and isoeugenol antifungal activities against *Candida albicans* strains (Minimum Inhibitory Concentrations—MICs).

Substances	MIC Range
ISO A1	1024 to > 1024 µg/mL
ISO A2	1024 to > 1024 µg/mL
ISO A3	1024 to > 1024 µg/mL
ISO A4	1024 to > 1024 µg/mL
ISO A5	1024 to > 1024 µg/mL
ISO A6	1024 to > 1024 µg/mL
ISO A7	1024 to > 1024 µg/mL
ISO A10	1024 to > 1024 µg/mL
ISO A11	1024 to > 1024 µg/mL
Isoeugenol	128 to 256 µg/mL

**Table 2:** Minimum Inhibitory Concentration (MIC) and Minimum Fungicidal Concentration (MFC) of isoeugenol and nystatin on *C. albicans*.

*C. albicans*	Isoeugenol (µg/mL)			Nystatin (µg/mL)			Controls	
	MIC	MFC	MFC/MIC	MIC	MFC	MFC/MIC	CB	FV
LM-4b	128	128	1	8	8	1	-	+
LM-5b	128	128	1	8	8	1	-	+
LM-10b	128	128	1	8	8	1	-	+
LM-11b	256	256	1	8	32	4	-	+
LM-13b	256	256	1	8	32	4	-	+
ATCC-76485	256	256	1	8	32	4	-	+

CB: culture broth, FV: fungal viability, (-) absence of fungal growth (+) presence of fungal growth.

**Table 3:** Association between isoeugenol and nystatin on *C. albicans* ATCC-76485 and LM-4b.

*C. albicans*	FIC		FICI	Effect
	FIC_A_	FIC_B_		
LM-4b	1.10	1.98	3.09	Indifference
ATCC-76485	0.98	1.12	2.10	Indifference

**Table 4:** Isoeugenol binding energies (kcal/mol) with selected fungal enzymes.

Fungal Enzymes	Isoeugenol Binding Energies (kcal/mol)
14-α-demethylase	−69.4023
Delta 14-sterol reductase	−84.5773
Lanosterol 14-α-demethylase	−34.0519
1,3-β-glucan synthase	−53.8295

## Supplementary Materials

Supplementary materials can be found at https://www.mdpi.com/1424-8247/13/10/291/s1.

## Abbreviations

ATCC	American Type Culture Collection
CFU	Colony Forming Units
DMSO	Dimethyl Sulfoxide
FICI	Fractional Inhibitory Concentration Index
FIC	Fractional Inhibitory Concentration
MFC	Minimum Fungicidal Concentration
MIC	Minimum Inhibitory Concentration
MVD	Molegro Virtual Docker
PDB	Protein Data Bank
RPMI	Roswell Park Memorial Institute
SDA	Sabouraud Dextrose Agar

## References

[1] Millsop J.W., Fazel N. (2016). Oral candidiasis. Clin. Dermatol..

[2] Colombo A.L., Guimarães T. (2003). Epidemiologia das infecções hematogênicas por *Candida* spp.. Rev. Soc. Bras. Med. Trop..

[3] Bassetti M., Merelli M., Righi E., Diaz-Martin A., Rosello E.M., Luzzari R., Parra A., Trecarichi E.M., Sanguinetti M., Posteraro B. (2013). Epidemiology, species distribution, antifungal susceptibility, and outcome of candidemia across five sites in Italy and Spain. J. Clin. Microbiol..

[4] Khan M.A.S., Ahmad I., Cameotra S.S. (2013). Phenyl aldehyde and propanoids exert multiple sites of action towards cell membrane and cell wall targeting ergosterol in *Candida albicans*. AMB Express.

[5] Yapar N. (2014). Epidemiology and risk factors for invasive candidiasis. Ther. Clin. Risk Manag..

[6] Castro R.D., Souza T.M.P.A., Bezerra L.M.D., Ferreira G.L.S., Costa E.M.M.B., Cavalcanti A.L. (2015). Antifungal activity and mode action of thymol and its synergism with nystatin against *Candida* species involved with infections in the oral cavity: An in vitro study. BMC Complement Altern. Med..

[7] Kalaiarasan K., Singh R., Chaturvedula L. (2018). Changing virulence factors amongs vaginal non-*albicans Candida* species. Indian J. Med. Microbiol..

[8] Mayer F.L., Wilson D., Hube B. (2013). *Candida albicans* pathogenecy mechanisms. Virulence.

[9] Williams D., Lewis M. (2011). Pathogenesis and treatment of oral candidoses. J. Oral. Microbiol..

[10] Sharma A. (2019). Oral candidiasis: An opportunistic infection: A review. Int. J. Applied Dent. Sci..

[11] Silva S., Rodrigues C.F., Araújo D., Rodrigues M.E., Henrique M. (2017). *Candida* species biofilms antifungal resistence. J. Fungi..

[12] Gárcia-Cuesta C., Sarrion-Pérez M.G., Bagán J.V. (2014). Current treatment of oral candidiasis: A literature review. J. Clin. Exp. Dent..

[13] Bassetti M., Peghin M., Timsit J.F. (2016). The current treatment landscape: Candidiasis. J. Antimicrob. Chem..

[14] Hipólito T.M.M., Bastos G.T.L., Barbosa T.W.L., Souza T.B., Coelho L.F.L., Dias A.L.T., Rodríguez I.C., Santos M.H., Dias D.F., Franco L.L. (2018). Synthesis, activity and docking studies of eugenol-based glucosides as new agents against *Candida* sp.. Chem. Biol. Drug Des..

[15] Zarlaha A., Kourkoumelis N., Stanojkovic T.P., Kovala-Demertri D. (2014). Cytotoxic activity of essential oil and extracts of *Ocimum basilicum* against human carcinoma cells. Molecular docking study of isoeugenol as a potent cox and lox inhibitor. Dig. J. Nanomater. Biostruct..

[16] Freire J.C.P., Júnior J.K.D.O., Silva D.D.F., Sousa J.P., Guerra F.Q.S., Lima E.O. (2017). Antifungal activity of essential oils against *Candida albicans* strains isolated from users of dental prostheses. Evid-Based Complement. Altern. Med..

[17] D’Souza S.P., Channavanar S.V., Kanchanashri B., Niveditha S.B. (2017). Pharmaceutical perspectives of spices and condiments as alternative antimicrobial remedy. Evid-Based Complement. Altern. Med..

[18] Afonso R.S., Rennó M.N., Slana G.B.C.A., França T.C.C. (2012). Aspectos químicos e biológicos do óleo essencial de cravo da Índia. Rev. Virtual Quím..

[19] Peixoto L.R., Rosalen P.L., Ferreira G.L.S., Freires I.A., Carvalho F.G., Castellano L.R., Castro R.D. (2017). Antifungal activity, mode of action and anti-biofilm effects of *Laurus nobilis* Linnaeus essential oil against *Candida* spp.. Arch. Oral Biol..

[20] Jukié M., Politeo O., Milos M. (2006). Chemical Composition and antioxidant effect of free volatile aglycones from nutmeg (*Myristica fragrans* Houtt.) compared to its essential oil. Croat. Chem. Acta.

[21] Nazzaro F., Fratianni F., Coppola R., Feo V.D. (2017). Essential oils and antifungal activity. Pharmaceuticals.

[22] Maia J.G.S., Andrade E.H.A. (2009). Database of the amazon aromatic plants and their essential oils. Quim. Nova.

[23] Gallucci M.N., Carezzano M.E., Oliva M.M., Demo M.S., Pizzolitto R.P., Zunino M.P., Zygadlo J.Á., Dambolema J.S. (2014). In vitro activity of natural phenolic compounds against fluconazole-resistant *Candida* species: A quantitative structure–activity relationship analysis. J. Appl. Microbiol..

[24] Kiessling A., Johansson D., Zahl I.H., Samuelsen O.B. (2009). Pharmacokinetics, plasma cortisol and effectiveness of benzocaine, MS-222 and isoeugenol measured in individual dorsal aorta-cannulated Atlantic salmon (Salmo salar). Aquaculture.

[25] Ruider S.A., Maulide N. (2015). Strong bonds made weak: Towards the general utility of amides as synthetic modules. Angew. Chem. Int. Ed..

[26] Katke S.A., Amrutkar S.V., Bhor R.J., Khairnar M.V. (2011). Synthesis of biologically active 2-chloro-N-alkyl/aryl acetamide derivatives. Int. J. Pharm. Sci. Res..

[27] Patel R.V., Kumari P., Rajani D.P., Chikhalia K.H. (2013). Synthesis of coumarin-based 1, 3, 4-oxadiazol-2ylthio-N-phenyl/benzothiazolyl acetamides as antimicrobial and antituberculosis agents. Med. Chem. Res..

[28] Jetti V., Chidurala P., Meshram J.S. (2015). Synthesis of new Acetamide-conjugated Monobactam antibiotics. Int. J. Pharm. Sci. Res..

[29] Aschale M. (2012). Synthesis and antimicrobial evaluation of some novel substituted 2-chloroacetanalides. Int. J. Chemtech. Res..

[30] Nielsen J. (2002). Combinatorial synthesis of natural products. Curr. Op. Chem. Biol..

[31] Cordeiro L., Figueiredo P., Souza H., Sousa A., Andrade-Júnior F., Medeiros D., Nóbrega J., Silva D., Martins E., Barbosa-Filho J. (2020). Terpinen-4-ol as an Antibacterial and Antibiofilm Agent against *Staphylococcus aureus*. Int. J. Mol. Sci..

[32] Strushkevich N., Usanov S.A., Park H.W. (2010). Structural basis of human CYP51 inhibition by antifungal azoles. J. Mol. Biol..

[33] Li X., Roberti R., Blobel G. (2015). Structure of an integral membrane sterol reductase from *Methylomicrobium alcaliphilum*. Nature.

[34] Monk B.C., Tomasiak T.M., Keniya M.V., Huschmann F.U., Tyndall J.D., O’Connell J.D., Cannon R.D., McDonald J.G., Rodriguez A., Finer-Moore J.S. (2014). Architecture of a single membrane spanning cytochrome P450 suggests constraints that orient the catalytic domain relative to a bilayer. Proc. Natl. Acad. Sci. USA.

[35] Garlatti V., Belloy N., Martin L., Lacroix M., Matsushita M., Endo Y., Fujita T., Camps J.C.F., Arlaud G.J., Thielens N.M. (2007). Structural insights into the innate immune recognition specificities of L-and H-ficolins. EMBO J..

[36] Bhatia R., Shreaz S., Khan N., Muralidhar S., Basir S.F., Manzoor N., Khan L.A. (2012). Proton pumping ATPase mediated fungicidal activity of two essential oil components. J. Basic Microbiol..

[37] Zemek J., Košiková B., Augustín J., Joniak D. (1979). Antibiotic properties of lignin components. Folia Microbiol..

[38] Pizzolito R.P., Barberis C.L., Dambolema J.S., Herrera J.M., Zunino M.P., Magnoli C.E., Rubinstein H.R., Zyglalo J., Dalcero A.M. (2015). Inhibitory effect of natural phenolic compounds on *Aspergillus parasiticus* growth. J. Chem..

[39] Laekeman G.M., Hoof L.V., Haermers A., Berghe D.A.V., Herman A.G., Vlietinck A.J. (1990). Eugenol a valuable compound for in vitro experimental research and worthwhile for further in vivo investigation. Phytother. Res..

[40] Siddique Z.N., Farooq F., Musthafa T.N.M., Ahmad A., Khan A.U. (2013). Synthesis, characterization and antimicrobial evaluation of novel halopyrazole derivatives. J. Saudi Chem. Soc..

[41] Dambolena J.S., López A.G., Meriles J.M., Rubistien H.R., Zygadlo J.A. (2012). Inhibitory effect of 10 natural phenolic compounds on Fusarium verticillioides. A structure- property- activity relationship study. Food Control.

[42] Abourashed E.A., Galal A.M., Shebl A.M., Mossa J.S. (2013). Enhancing effect of isoeugenol on the antimicrobial activity of isoniazid, 6-paradol and 6- shogaol. J. Herbs Spices Med. Plants.

[43] Clinical Laboratory Standards Institute (CLSI) (2002). Reference Method for Broth Dilution Antifungal Susceptibility Testing of Yeasts.

[44] Silva D., Diniz-Neto H., Cordeiro L., Silva-Neta M., Silva S., Andrade-Júnior F., Leite M., Nóbrega J., Morais M., Souza J. (2020). (R)-(+)-Citronellol and (S)-(-)-Citronellol in Combination with Amphotericin B against *Candida* spp.. Int. J. Mol. Sci..

[45] Hadacek F., Greger H. (2000). Testing of antifungal natural products: Methodologies, comparability of results and assay choice. Phytochem. Analysis.

[46] Morales G., Paredes A., Sierra P., Loyola L.A. (2008). Antimicrobial activity of three baccharis species used in the tradicional medicine of Northern Chile. Molecules.

[47] Shin S. (2003). Anti-*Aspergillus* activities of plant essential oils and their combination effects with-ketoconazole or amphotericin b. Arch. Pharm. Res..

[48] Han X., Wang N., Li J., Wang Y., Wang R., Chang J. (2019). Identification of nafamostat mesilate as an inhibitor of the fat mass and obesity-associated protein (FTO) demethylase activity. Chem. Biol. Interact..

