# Supplementary material for: Isoeugenol and Hybrid Acetamides against Candida albicans Isolated from the Oral Cavity
Source: Pharmaceuticals (Basel). 2020 Oct 3;13(10):291. doi: 10.3390/ph13100291 (PMC7599878; doi:10.3390/ph13100291)
Supplement: Supplementary file 1 [file pharmaceuticals-13-00291-s001.pdf]

pharmaceuticals

Article

Isoeugenol and hybrid acetamides against *Candida albicans* isolated from the oral cavity

Daianne Medeiros [1], José Oliveira-Júnior [1], Jefferson Nóbrega [1], Laísa Cordeiro [1], Jeane Jardim [2], Helivaldo Souza [2]*, Gracielle Silva [2], Petrônio Athayde-Filho [2], José Barbosa-Filho [1], Luciana Scotti [1], Edeltrudes Lima [1]

[1] Department of Pharmaceutical Science, Health Sciences Center, Federal University of Paraíba, 58033-455, João Pessoa, Paraíba, Brazil; daiannemedeiros1@gmail.com (D.M.); joseklidemberg@gmail.com (J.O-J.); jeffersonrodriguesn@hotmail.com (J.N.); laisavilar@gmail.com (L.C.); jbarbosa@ltf.ufpb.br (J.B-F).; luciana.scotti@gmail.com (L.S.); edelolima@yahoo.com.br (E.L.)

[2] Chemistry Department, Exact and Natural Sciences Center, Federal University of Paraíba, 58033-455, João Pessoa, Brazil; jeanegalindo@hotmail.com (J.J.); helivaldog3@gmail.com (H.S.); gracielletavares@ltf.ufpb.br (G.S.); athayde-filho@quimica.ufpb.br (P.A-F.)

* Correspondence: helivaldog3@gmail.com; Tel.: +55 83 3216-7347 (H.S.)

Supplementary Material

2.1. Chemistry

All reagents and solvents were purchased from commercial sources Sigma-Aldrich (São Paulo, Brazil). The recrystallization technique was used to purify the compound and its purity was confirmed from the melting point measurement using the MQAPF-302 (Microquímica) apparatus. The hydrogen and carbon nuclear magnetic resonance (NMR) spectra were obtained from a Bruker Avance 400 apparatus. The hydrogen spectra (^1H) were obtained at the frequency of 400 and 500 MHz using deuterated solvents chloroform ($CDCl_3$). The carbon spectra (^{13}C) were obtained at the frequency of 101 MHz using deuterated solvents chloroform ($CDCl_3$). The chemical distances (δ) were generated in parts per million (ppm) and in hertz (Hz) the coupling constants (*J*) were determined. High Resolution Mass (HRMS) analysis was obtained with time-of-flight (TOF) mass spectrometer (microTOFII - Bruker) instrument using Electron Impact Ionization (EI) and reported as *m/z* (relative intensity) for the molecular ion [M] and reporting the molecular ion [M+H].

2.2. General procedure for the preparation of isoeugenol derivatives

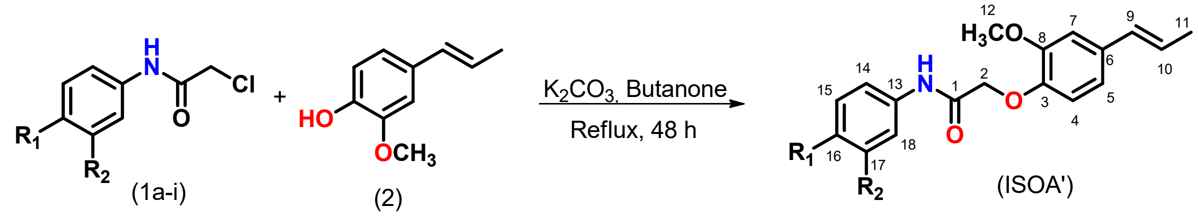

(**ISOA1**): R_1 = H; R_2 = H
(**ISOA2**): R_1 = NO_2; R_2 = H
(**ISOA3**): R_1 = CH_2CH_3; R_2 = H
(**ISOA4**): R_1 = CH_3; R_2 = H
(**ISOA5**): R_1 = Br; R_2 = H
(**ISOA6**): R_1 = Cl; R_2 = H
(**ISOA7**): R_1 = $CH(CH_3)_2$; R_2 = H
(**ISOA10**): R_1 = Cl; R_2 = NO_2
(**ISOA11**): R_1 = OCH_3; R_2 = H

In a 50 mL flask, a mixture of isoeugenol (1.1 eq), K_2CO_3 (2.0 eq) solubilized in 10 mL of butanone was stirred at room temperature for 1 h. 2-chloro-*N*-arylacetamides[1] (1.0 eq) was added and the reaction mixture was refluxed for 48 h. At the end of the reaction, the mixture was cooled and water (30 mL) was added, extracted with ethyl acetate (2 x 30 mL), washed with water (2 x 30 mL), saturated sodium chloride solution (30 mL), dried over sodium sulfate and then evaporated under reduced pressure. The products were recrystallized using ethanol as the solvent.

(*E*)-2-(2-methoxy-4-(prop-1-en-1-yl)phenoxy)-*N*-phenylacetamide (**ISOA1**): Yield: 76%, m.p.: 80-82 °C. ^{1}H NMR (400 MHz, $CDCl_3$) δ 8.92 (s, 1H, N-H), 7.59 (d, *J* = 8.0 Hz, 2H, H-14 and H-18), 7.34 (t, *J* = 7.9 Hz, 2H, H-15 and H-17), 7.13 (t, *J* = 7.4 Hz, 1H, H-16), 6.90 (t, *J* = 7.7 Hz, 3H, H-4, H-5 and H-7), 6.34 (d, *J* = 16.0 Hz, 1H, H-9), 6.15 (dq, *J* = 13.1, 6.6 Hz, 1H, H-10), 4.63 (s, 2H, H-2), 3.94 (s, 3H, H-12), 1.87 (d, *J* = 6.6 Hz, 3H, H-11). ^{13}C NMR (101 MHz, $CDCl_3$) δ 166.96 (C-1), 149.82 (C-2), 146.27 (C-8), 137.28 (C-13), 133.97 (C-6), 130.25 (C-7), 129.05 (C-15 and C-17), 125.38 (C-4), 124.56 (C-16), 119.87 (C-14 and C-18), 119.00 (C-5), 116.63 (C-9), 109.32 (C-10), 70.63 (C-2), 55.89 (C-12), 18.39 (C-11). HRMS *m*/*z*, calculated for $C_{18}H_{19}NO_3$ [M + H] = 298.1443, found: 298.1438.

(*E*)-2-(2-methoxy-4-(prop-1-en-1-yl)phenoxy)-*N*-(4-nitrophenyl)acetamide (**ISOA2**): Yield: 80%, m.p.: 120-122 °C. ^{1}H NMR (400 MHz, $CDCl_3$) δ 9.36 (s, 1H, N-H), 8.23 (d, *J* = 9.0 Hz, 2H, H-15 and H-17), 7.79 (d, *J* = 8.9 Hz, 2H, H-14 and H-18), 6.93 (d, *J* = 13.1 Hz, 3H, H-4, H-5 and H-7), 6.35 (d, *J* = 15.7 Hz, 1H, H-9), 6.17 (dq, *J* = 13.3, 6.5 Hz, 1H, H-10), 4.66 (s, 2H, H-2), 3.96 (s, 3H, H-12), 1.88 (d, *J* = 6.6 Hz, 3H, H-11). ^{13}C NMR (101 MHz, $CDCl_3$) δ 167.68 (C-1), 149.81 (C-3), 146.06 (C-8), 143.81 (C-16), 143.08 (C-13), 134.48 (C-6), 130.11 (C-7), 125.78 (C-4), 125.13 (C-15 and C-17), 119.23 (C-14 and C-18), 119.18 (C-5), 117.19 (C-9), 109.55 (C-10), 70.87 (C-2), 56.05 (C-12), 18.41 (C-11). HRMS *m*/*z*, calculated for $C_{18}H_{18}N_2O_5$ [M + H] = 343.1294, found: 343.1274

(*E*)-*N*-(4-ethylphenyl)-2-(2-methoxy-4-(prop-1-en-1-yl)phenoxy)acetamide (**ISOA3**): Yield: 92%, m.p.: 83-85 °C. ^{1}H NMR (400 MHz, $CDCl_3$) δ 8.85 (s, 1H, N-H), 7.49 (d, *J* = 8.3 Hz, 2H, H-14 and H-18), 7.17 (d, *J* = 8.3 Hz, 2H, H-15 and H-17), 6.94 – 6.88 (m, 3H, H-4, H-5 and H-7), 6.34 (d, *J* = 15.7 Hz, 1H, H-9), 6.15 (dq, *J* = 13.2, 6.6 Hz, 1H, H-10), 4.62 (s, 2H, H-2), 3.94 (s, 3H, H-12), 2.62 (q, *J* = 7.6 Hz, 2H,

CH_2), 1.87 (d, *J* = 6.5 Hz, 3H, H-11), 1.22 (t, *J* = 7.6 Hz, 3H, CH_3). ^{13}C NMR (101 MHz, $CDCl_3$) δ 166.81 (C-1), 149.82 (C-3), 146.30 (C-8), 140.69 (C-16), 134.87 (C-13), 133.91 (C-6), 130.26 (C-7), 128.38 (C-14 and C-18), 125.34 (C-4), 120.04 (C-15 and C-17), 118.99 (C-5), 116.56 (C-9), 109.30 (C-10), 70.61 (C-2), 55.89 (C-12), 28.34 (CH_2), 18.40 (C-11), 15.66 (CH_3). HRMS *m*/*z*, calculated for $C_{20}H_{23}NO_3$ [M + H] = 326.1756, found: 326.1750.

(*E*)-2-(2-methoxy-4-(prop-1-en-1-yl)phenoxy)-*N*-(p-tolyl)acetamide (**ISOA4**): Yield: 84%, m.p.: 81-83 °C. ^{1}H NMR (400 MHz, $CDCl_3$) δ 8.86 (s, 1H, N-H), 7.48 (d, *J* = 8.3 Hz, 2H, H-14 and H-18), 7.15 (d, *J* = 8.2 Hz, 2H, H-15 and H-17), 6.91 (d, *J* = 14.4 Hz, 3H, H-4, H-5 and H-7), 6.35 (dd, *J* = 15.7, 1.5 Hz, 1H, H-9), 6.16 (dq, *J* = 13.2, 6.6 Hz, 1H, H-10), 4.62 (s, 2H, H-2), 3.94 (s, 3H, H-12), 2.33 (s, 3H, CH_3), 1.88 (dd, *J* = 6.6, 1.5 Hz, 3H, H-11). ^{13}C NMR (101 MHz, $CDCl_3$) δ 166.83 (C-1), 149.84 (C-3), 146.31 (C-8), 134.75 (C-13), 134.22 (C-16), 133.91 (C-6), 130.29 (C-7), 129.56 (C-14 and C-18), 125.34 (C-4), 119.95 (C-15 and C-17), 119.00 (C-5), 116.57 (C-9), 109.32 (C-10), 70.61 (C-2), 55.90 (C-12), 20.92 (CH_3), 18.42 (C-11). HRMS *m*/*z*, calculated for $C_{19}H_{21}NO_3$ [M + H] = 312.1600, found: 312.1585.

(*E*)-*N*-(4-bromophenyl)-2-(2-methoxy-4-(prop-1-en-1-yl)phenoxy)acetamide (**ISOA5**): Yield: 72%, m.p.: 103-105 °C. ^{1}H NMR (400 MHz, $CDCl_3$) δ 8.97 (s, 1H, N-H), 7.50 (d, *J* = 9.1 Hz, 2H, H-15 and H-17), 7.44 (d, *J* = 9.0 Hz, 2H, H-14 and H-18), 6.90 (t, *J* = 8.0 Hz, 3H, H4, H-5, H-7), 6.34 (dd, *J* = 15.7, 1.6 Hz, 1H, H-9), 6.15 (dq, *J* = 13.2, 6.6 Hz, 1H, H-10), 4.61 (s, 2H, H-2), 3.93 (s, 3H, H-12), 1.87 (dd, *J* = 6.6, 1.7 Hz, 3H, H-11). ^{13}C NMR (101 MHz, $CDCl_3$) δ 167.05 (C-1), 149.79 (C-3), 146.17 (C-8), 136.39 (C-13), 134.12 (C-6), 132.02 (C-14 and C-18), 130.19 (C-7), 125.51 (C-4), 121.36 (C-15 and C-17), 119.05 (C-5), 117.14 (C-16), 116.80 (C-9), 109.38 (C-10), 70.68 (C-2), 55.93 (C-12), 18.40 (C-11). HRMS *m*/*z*, calculated for $C_{18}H_{18}BrNO_3$ [M + H] = 376.0548, found: 376.0526.

(*E*)-*N*-(4-chlorophenyl)-2-(2-methoxy-4-(prop-1-en-1-yl)phenoxy)acetamide (**ISOA6**): Yield: 90%, m.p.: 92-94 °C. ^{1}H NMR (400 MHz, $CDCl_3$) δ 8.96 (s, 1H, N-H), 7.55 (d, *J* = 8.9 Hz, 2H, H-15 and H-17), 7.30 (d, *J* = 8.8 Hz, 2H, H-14 and H-18), 6.91 (t, *J* = 7.8 Hz, 3H, H-4, H-5 and H-7), 6.34 (dd, *J* = 15.7, 1.6 Hz, 1H, H-9), 6.16 (dq, *J* = 15.7, 6.6 Hz, 1H, H-10), 4.62 (s, 2H, H-2), 3.94 (s, 3H, H-12), 1.88 (dd, *J* = 6.6, 1.6 Hz, 3H, H-11). ^{13}C NMR (101 MHz, $CDCl_3$) δ 167.03 (C-1), 149.81 (C-3), 146.19 (C-8), 135.89 (C-13), 134.13 (C-6), 130.20 (C-7), 129.54 (C-16), 129.08 (C-14 and C-18), 125.52 (C-4), 121.04 (C-15 and C-17), 119.06 (C-5), 116.80 (C-9), 109.39 (C-10), 70.67 (C-2), 55.94 (C-12), 18.40 (C-11). HRMS *m*/*z*, calculated for $C_{18}H_{18}ClNO_3$ [M + H] = 332.1053, found: 332.1036.

(*E*)-*N*-(4-isopropylphenyl)-2-(2-methoxy-4-(prop-1-en-1-yl)phenoxy)acetamide (**ISOA7**): Yield: 76%, m.p.: 98-100 °C. ^{1}H NMR (400 MHz, $CDCl_3$) δ 8.85 (s, 1H, N-H), 7.49 (d, *J* = 8.6 Hz, 2H, H-14 and H-18), 7.20 (d, *J* = 8.4 Hz, 2H, H-15 and H-17), 6.93 – 6.88 (m, 3H, H-4, H-5 and H-7), 6.34 (dd, *J* = 15.7, 1.7 Hz, 1H, H-9), 6.15 (dq, *J* = 15.7, 6.6 Hz, 1H, H-10), 4.62 (s, 3H, H-2), 3.94 (s, 3H, H-12), 2.88 (hept, *J* = 6.9 Hz, 1H, CH), 1.87 (dd, *J* = 6.6, 1.6 Hz, 3H, H-11), 1.23 (d, *J* = 6.9 Hz, 6H, CH_3). ^{13}C NMR (101 MHz, $CDCl_3$) δ 166.83 (C-1), 149.81 (C-3), 146.30 (C-8), 145.32 (C-16), 134.92 (C-13), 133.90 (C-6), 130.27 (C-7), 126.94 (C-14 and C-18), 125.33 (C-4), 120.06 (C-15 and C-17), 118.99 (C-5), 116.55 (C-9), 109.30 (C-10), 70.61 (C-2), 55.88 (C-12), 33.63 (CH), 24.02 (CH_3), 18.39 (C-11). HRMS *m*/*z*, calculated for $C_{21}H_{25}NO_3$ [M + H] = 340.1913, found: 340.1899.

(*E*)-*N*-(4-chloro-3-nitrophenyl)-2-(2-methoxy-4-(prop-1-en-1-yl)phenoxy)acetamide (**ISOA10**): Yield: 72%, m.p.: 118-120 °C. ^{1}H NMR (400 MHz, $CDCl_3$) δ 9.31 (s, 1H, N-H), 8.20 (d, *J* = 2.5 Hz, 1H, H-18), 7.81 (dd, *J* = 8.8, 2.4 Hz, 1H, H-14), 7.48 (d, *J* = 8.8 Hz, 1H, H-15), 6.91 (d, *J* = 15.7 Hz, 3H, H-4, H-5 and H-7), 6.34 (dd, *J* = 15.7, 1.6 Hz, 1H, H-9), 6.16 (dq, *J* = 15.7, 6.5 Hz, 1H, H-10), 4.63 (s, 2H, H-2), 3.95 (s, 3H, H-12), 1.87 (dd, *J* = 6.6, 1.6 Hz, 3H, H-11). ^{13}C NMR (101 MHz, $CDCl_3$) δ 167.73 (C-1), 149.84 (C-3), 147.96 (C-17), 146.13 (C-8), 137.04 (C-13), 134.57 (C-6), 132.34 (C-14), 130.18 (C-7), 125.89 (C-, 123.95 (C-15), 121.75 (C-16), 119.28 (C-5), 117.33 (C-4), 116.46 (C-9), 109.63 (C-10), 70.96 (C-2), 56.14 (C-12), 18.50 (C-11). HRMS *m*/*z*, calculated for $C_{18}H_{17}ClN_2O_5$ [M + H] = 377.0904, found: 377.0881.

(*E*)-2-(2-methoxy-4-(prop-1-en-1-yl)phenoxy)-*N*-(4-methoxyphenyl)acetamide (**ISOA11**): Yield: 80%, m.p.: 96-98 °C. ^{1}H NMR (400 MHz, $CDCl_3$) δ 8.80 (s, 1H, N-H), 7.49 (d, *J* = 9.0 Hz, 2H, H-14 and H-18), 6.94 – 6.86 (m, 5H, H-15, H-17, H-4, H-5 and H-7), 6.34 (dd, *J* = 15.7, 1.6 Hz, 1H, H-9), 6.15 (dq, *J* = 15.7, 6.6 Hz, 1H, H-10), 4.62 (s, 2H, H-2), 3.94 (s, 3H, H-12), 3.79 (s, 3H, OCH_3), 1.88 (dd, *J* = 6.6, 1.6 Hz, 3H, H-11). ^{13}C NMR (101 MHz, $CDCl_3$) δ 166.68 (C-1), 156.61 (C-16), 149.80 (C-3), 146.29 (C-8), 133.88 (C-6), 130.40 (C-13), 130.26 (C-7), 125.34 (C-14 and C-18), 121.64 (C-4), 118.99 (C-5), 116.49 (C-9), 114.21 (C-15 and C-17), 109.30 (C-10), 70.53 (C-2), 55.89 (OCH_3), 55.48 (C-12), 18.40 (C-11). HRMS *m*/*z*, calculated for $C_{19}H_{21}NO_4$ [M + H] = 328.1549, found: 328.1543.

2.3. NMR and Mass Spectrum

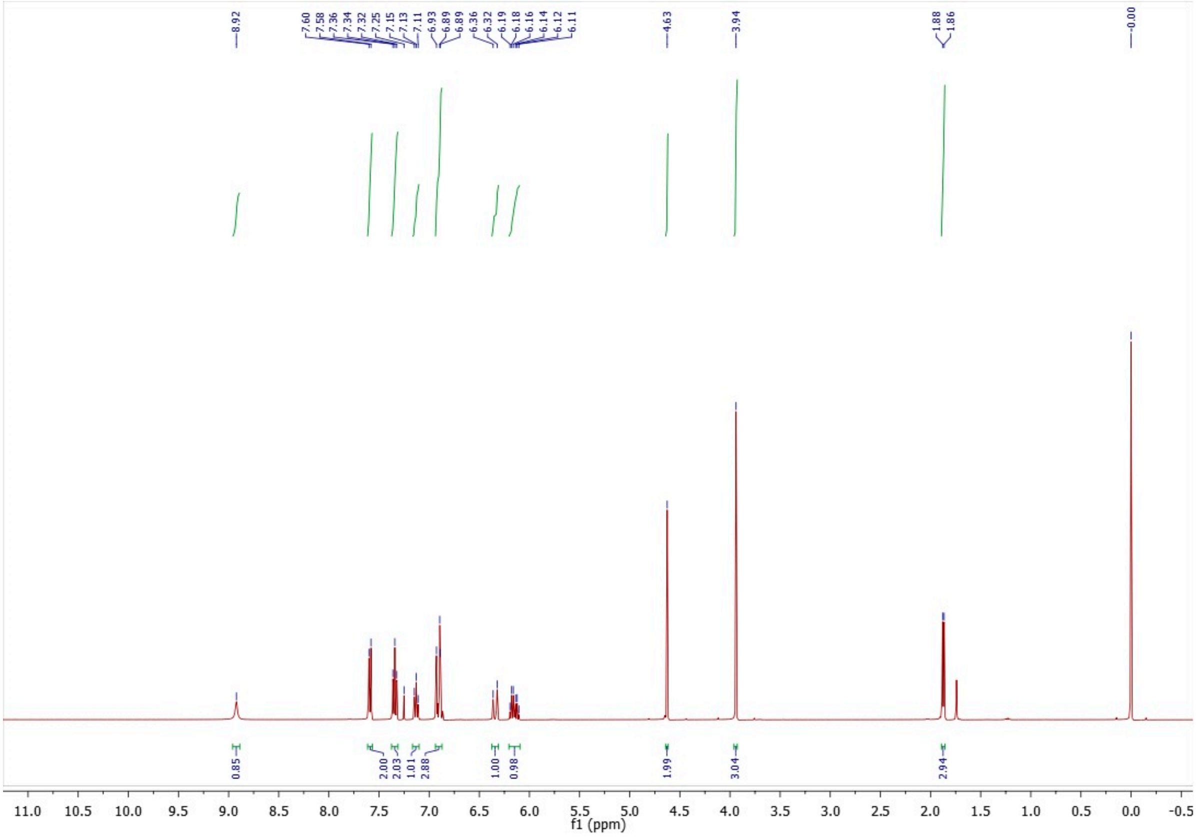

Figure S1: ^{1}H NMR spectrum (400 MHz, $CDCl_3$) of compound ISOA1.

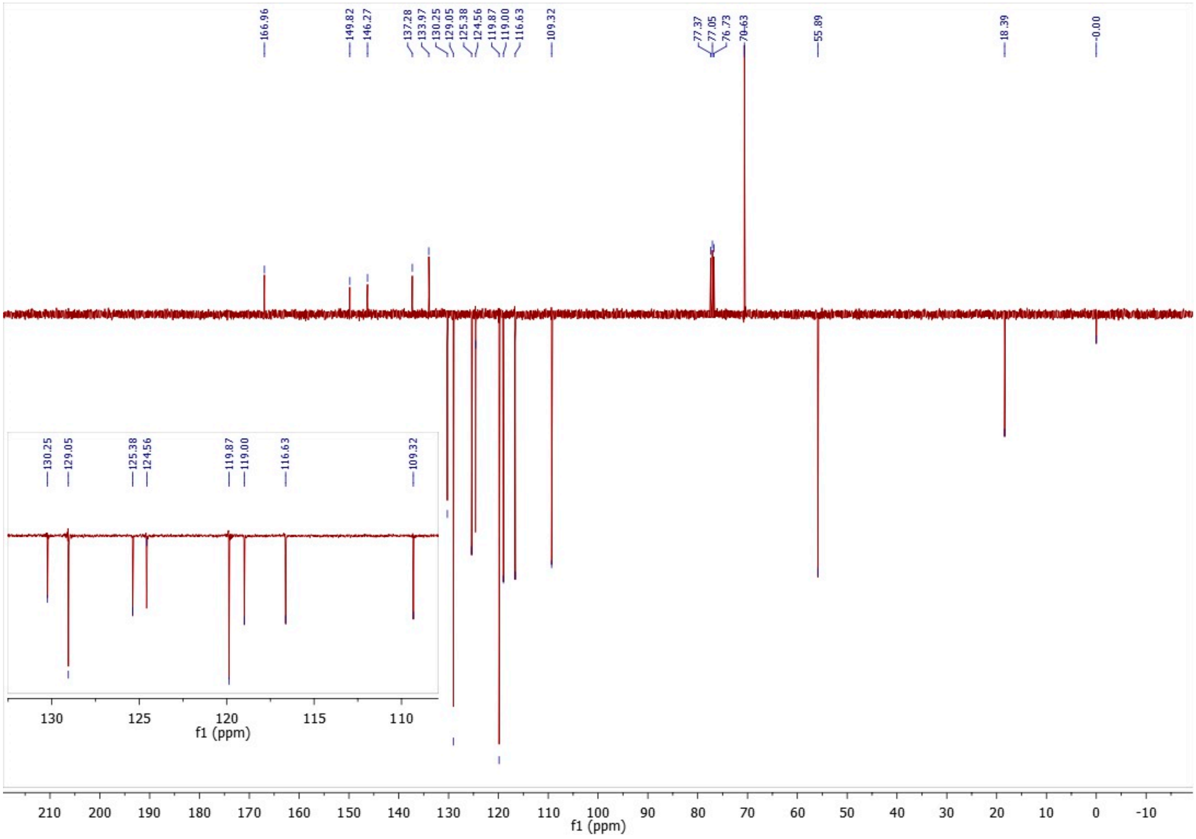

Figure S2: ^{13}C NMR spectrum (101 MHz, $CDCl_3$) of compound ISOA1.

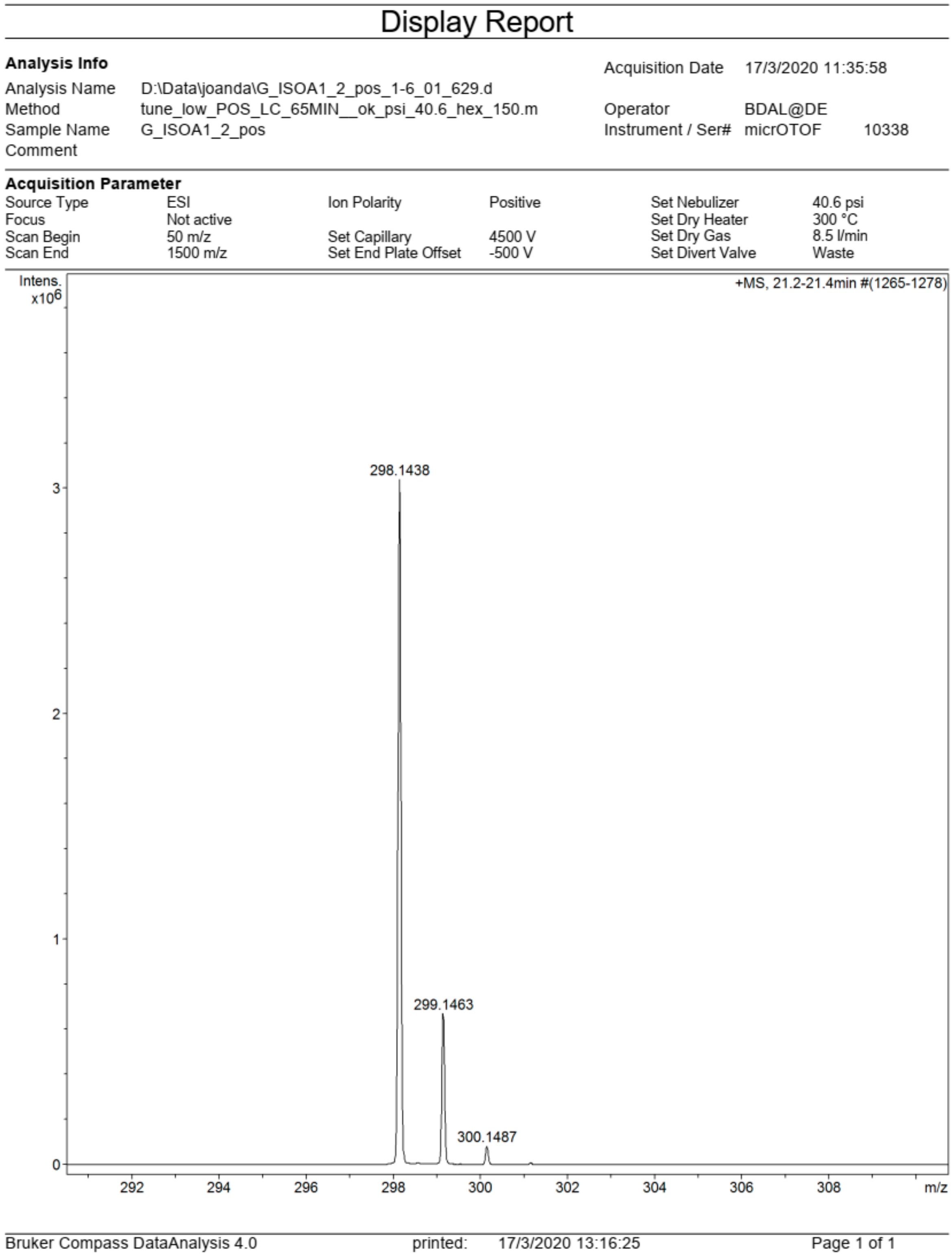

Figure S3: Mass spectrum of compound ISOA1.

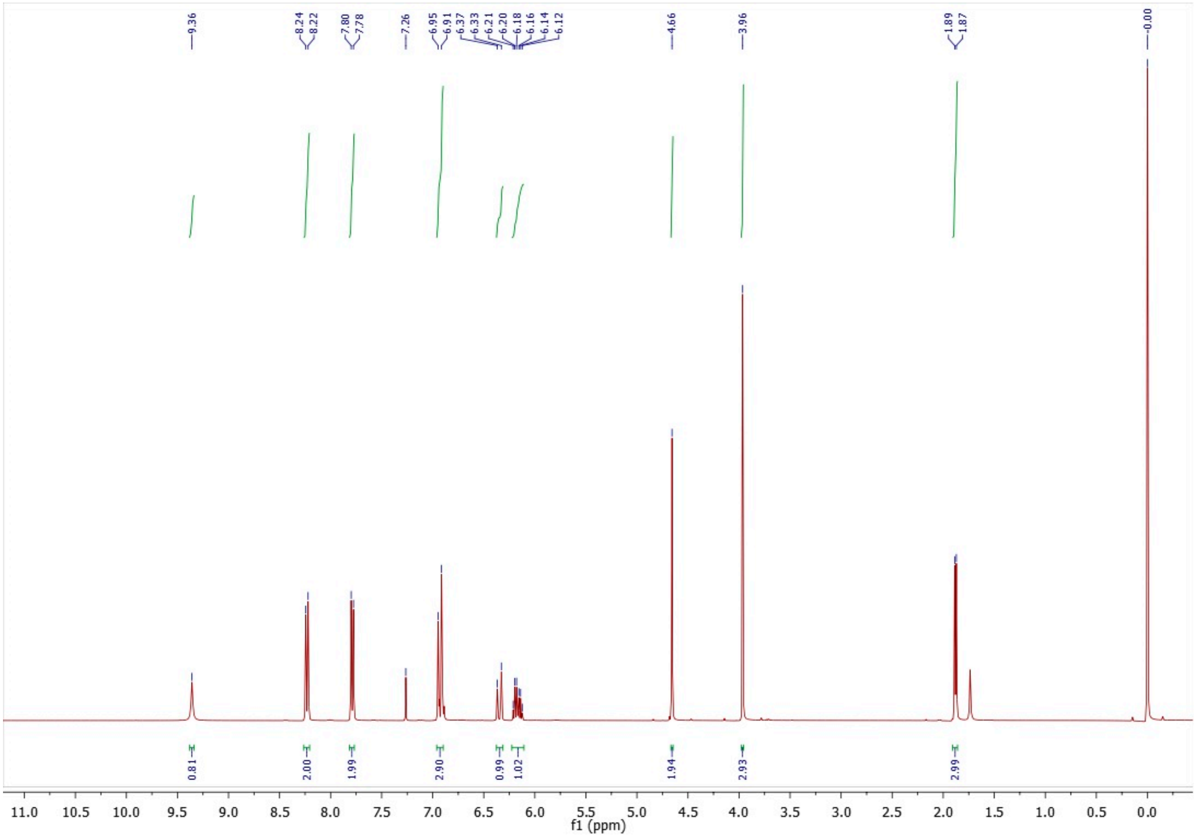

Figure S4: ^{1}H NMR spectrum (400 MHz, $CDCl_3$) of compound ISOA2.

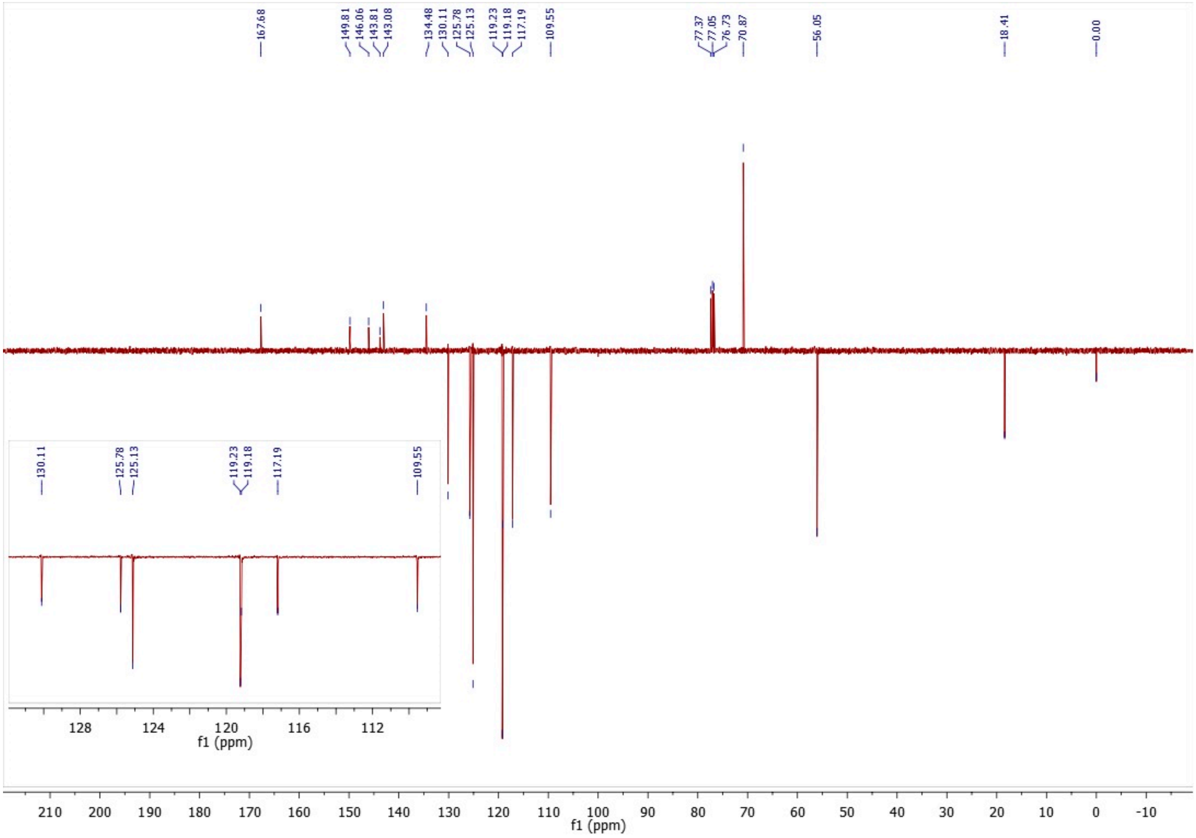

Figure S5: ^{13}C NMR spectrum (101 MHz, $CDCl_3$) of compound ISOA2.

Display Report

Analysis Info

Analysis Name	D:\Data\Prof Barbosa\ISA000002_11.02.19.d	Acquisition Date	2/11/2019 4:25:14 PM
Method	tune_wide_POS_ID_Lucas.m	Operator	BDAL@DE
Sample Name		Instrument / Ser#	micrOTOF 10338
Comment	ISA000002<br>MODO POSITIVO<br>11.02.19		

Acquisition Parameter

Source Type	ESI	Ion Polarity	Positive	Set Nebulizer	5.8 psi
Focus	Not active			Set Dry Heater	180 °C
Scan Begin	50 m/z	Set Capillary	4500 V	Set Dry Gas	4.0 l/min
Scan End	3000 m/z	Set End Plate Offset	-500 V	Set Divert Valve	Waste

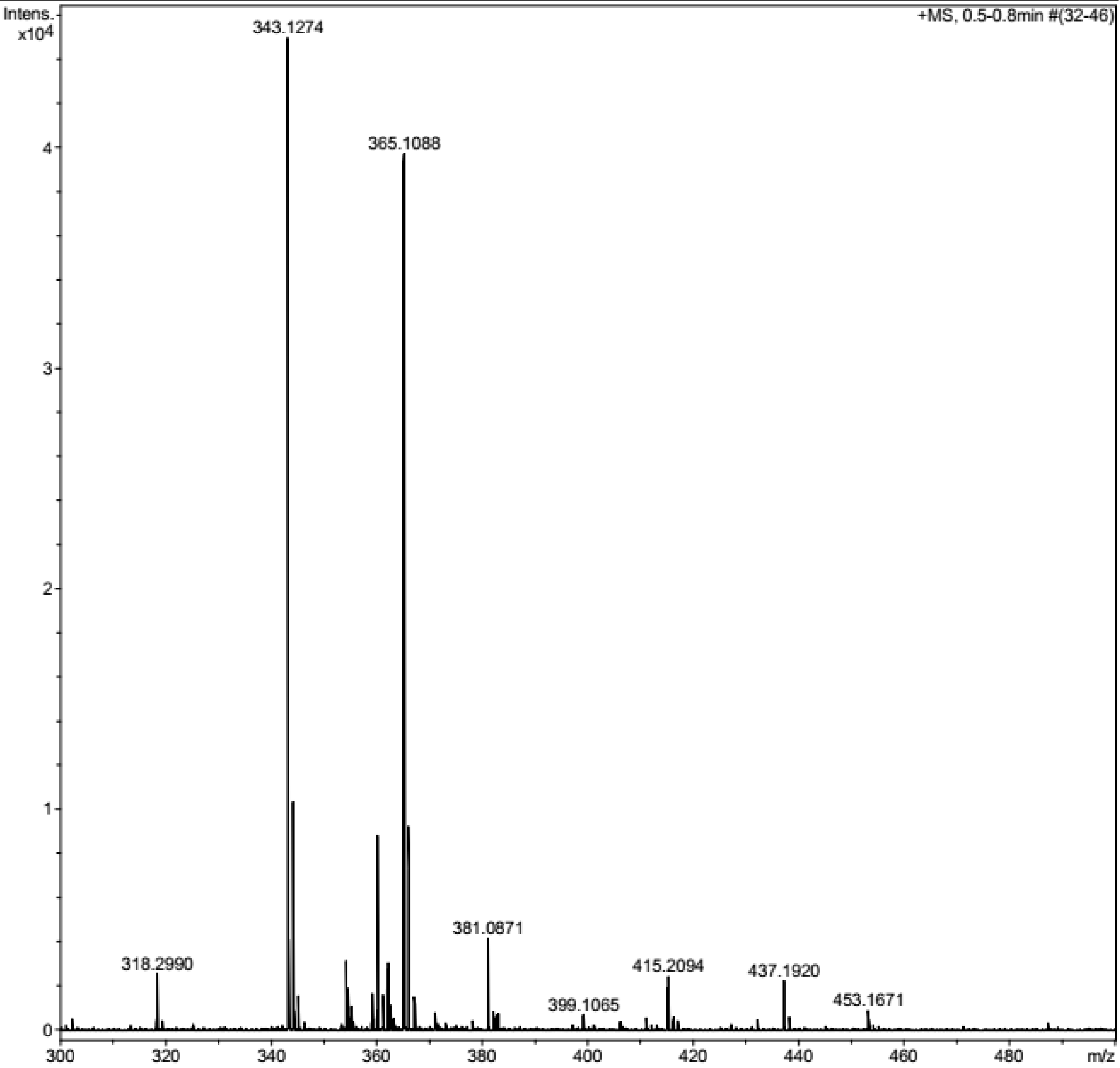

Bruker Compass DataAnalysis 4.0 printed: 2/11/2019 6:23:07 PM Page 1 of 1

Figure S6: Mass spectrum of compound ISOA2.

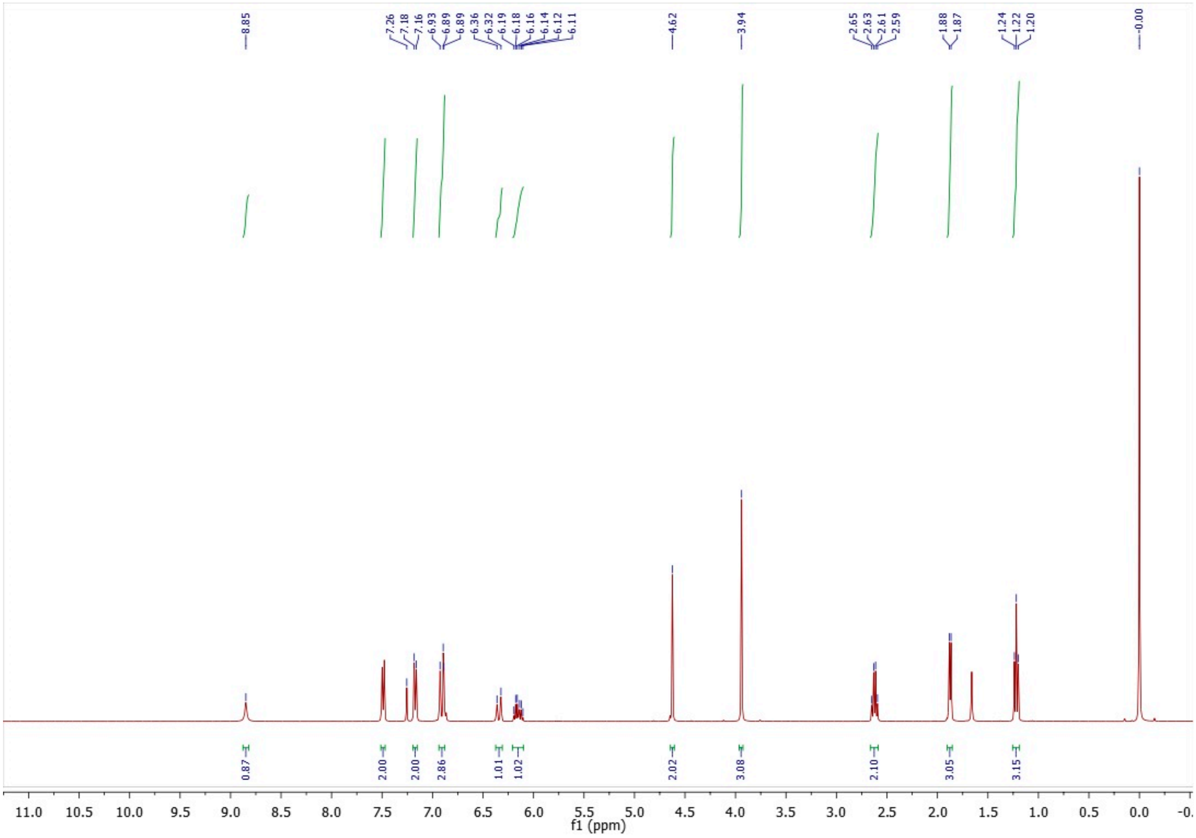

Figure S7: ^{1}H NMR spectrum (400 MHz, $CDCl_3$) of compound ISOA3.

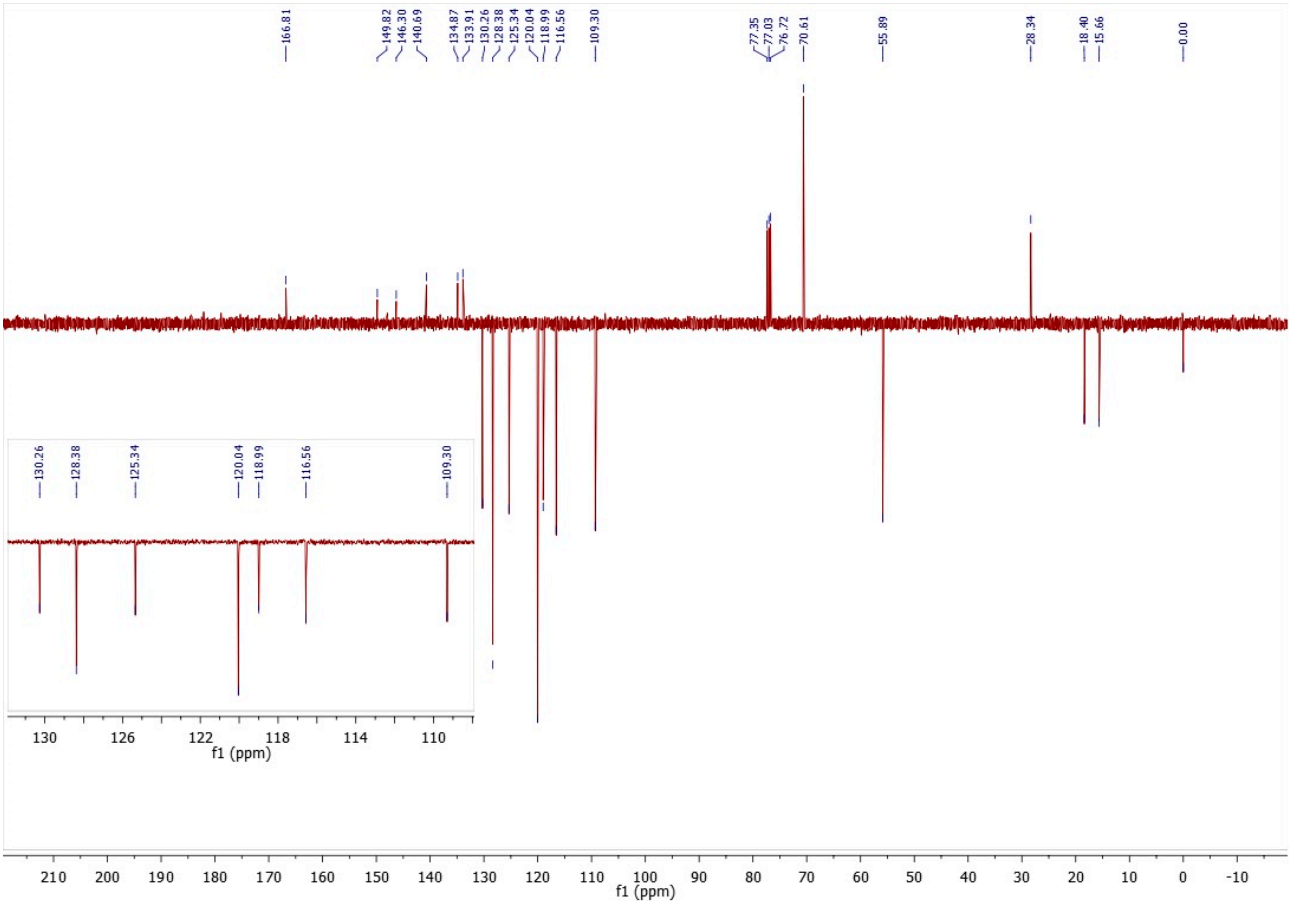

Figure S8: ^{13}C NMR spectrum (101 MHz, $CDCl_3$) of compound ISOA3.

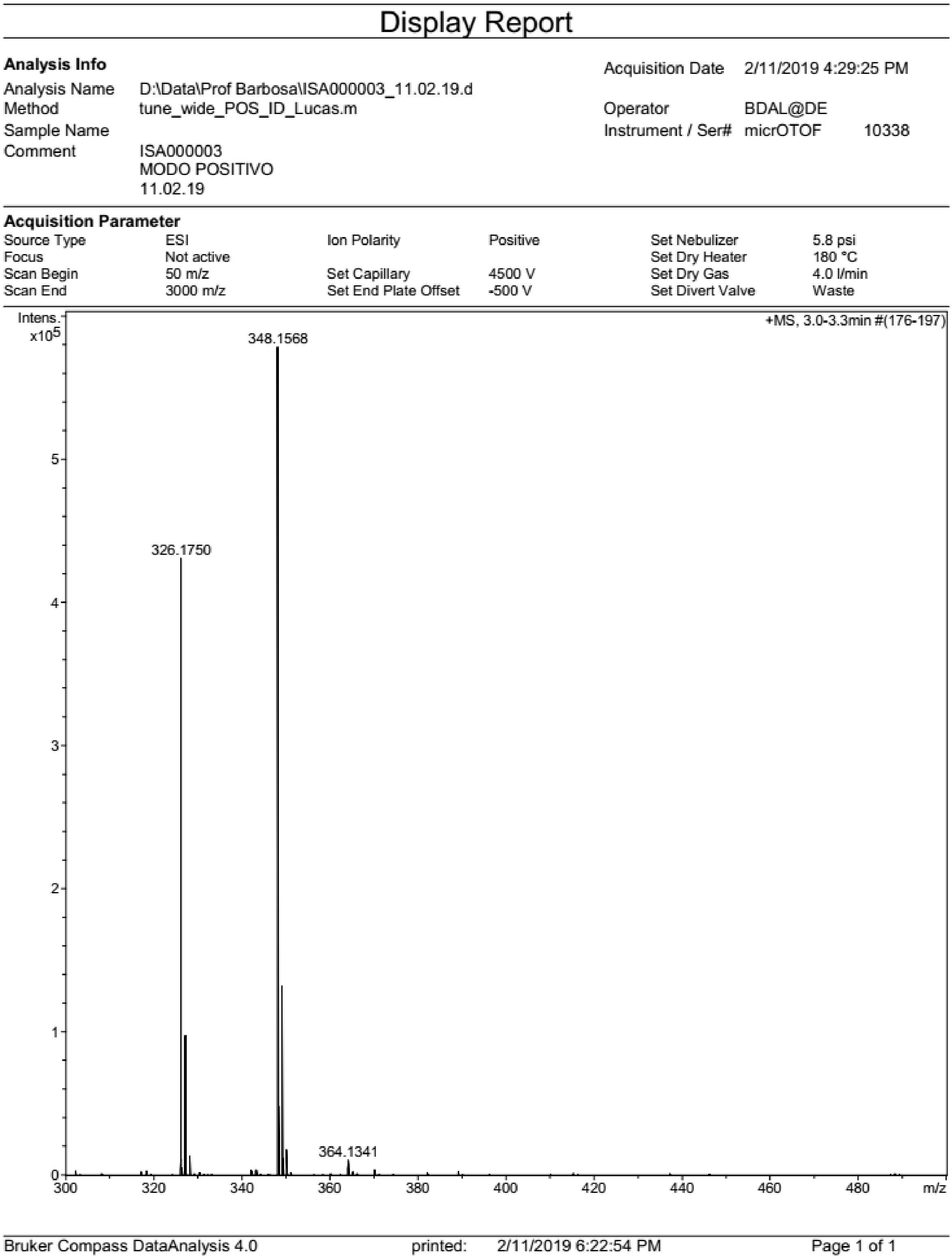

Figure S9: Mass spectrum of compound ISOA3.

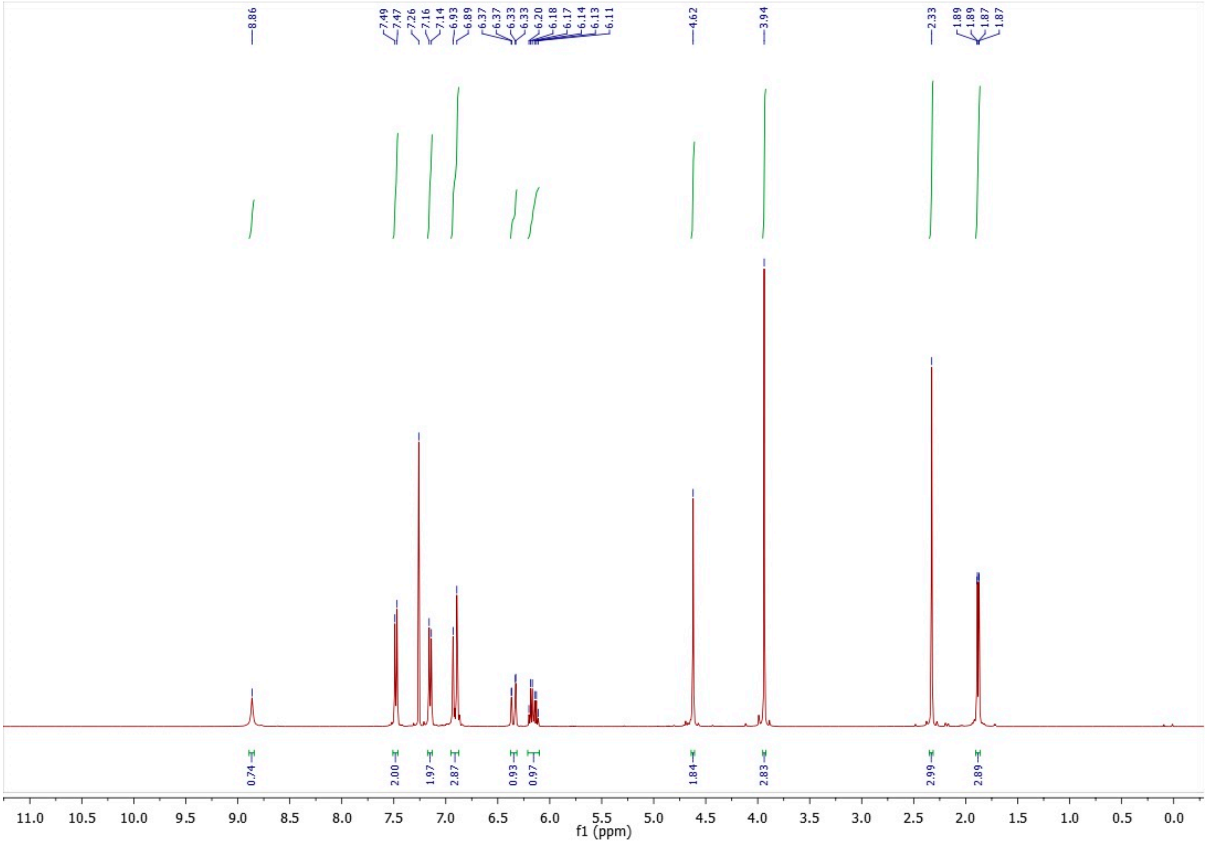

Figure S10: ^{1}H NMR spectrum (400 MHz, $CDCl_3$) of compound ISOA4.

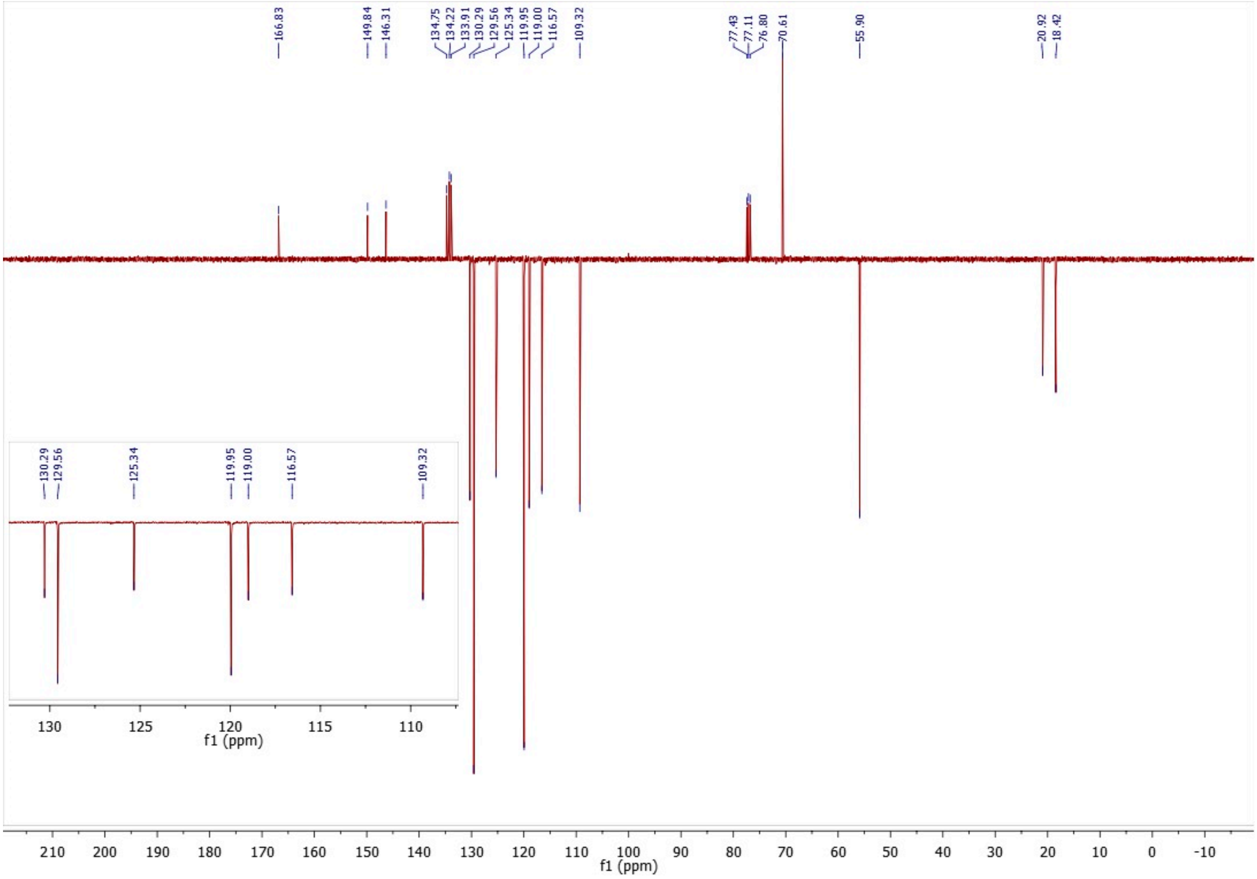

Figure S11: ^{13}C NMR spectrum (101 MHz, $CDCl_3$) of compound ISOA4.

Display Report

Analysis Info

Analysis Name	D:\Data\joanda\G_ISOA4_4_pos_1-7_01_640.d	Acquisition Date	17/3/2020 21:12:46
Method	tune_low_POS_LC_15MIN_tunemix_ok_psi_40.6_hex_150.m	Operator	BDAL@DE
Sample Name	G_ISOA4_4_pos	Instrument / Ser#	micrOTOF 10338
Comment			

Acquisition Parameter

Source Type	ESI	Ion Polarity	Positive	Set Nebulizer	40.6 psi
Focus	Not active			Set Dry Heater	300 °C
Scan Begin	50 m/z	Set Capillary	4500 V	Set Dry Gas	8.5 l/min
Scan End	3000 m/z	Set End Plate Offset	-500 V	Set Divert Valve	Waste

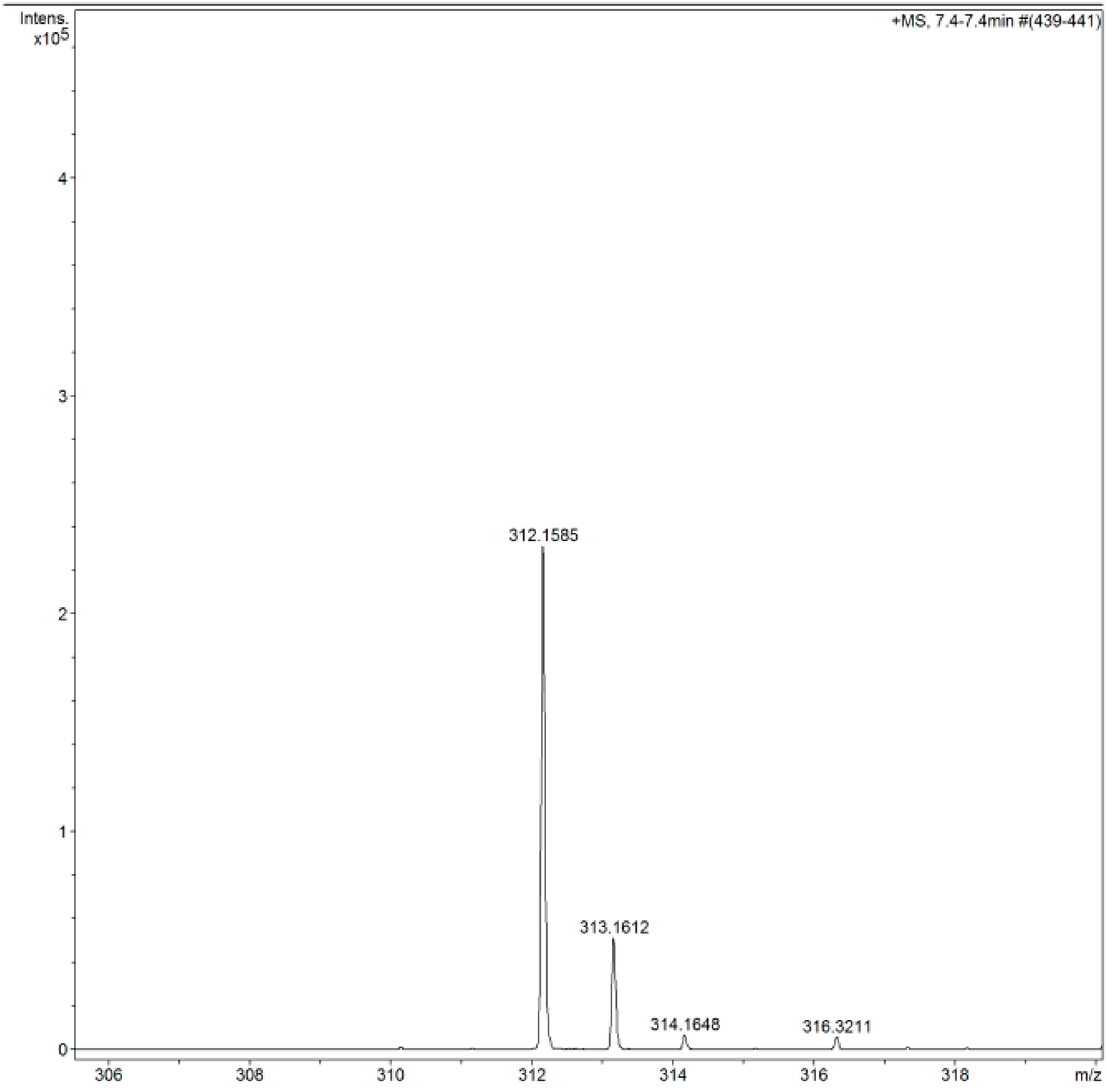

Bruker Compass DataAnalysis 4.0 printed: 17/3/2020 21:35:56 Page 1 of 1

Figure S12: Mass spectrum of compound ISOA4.

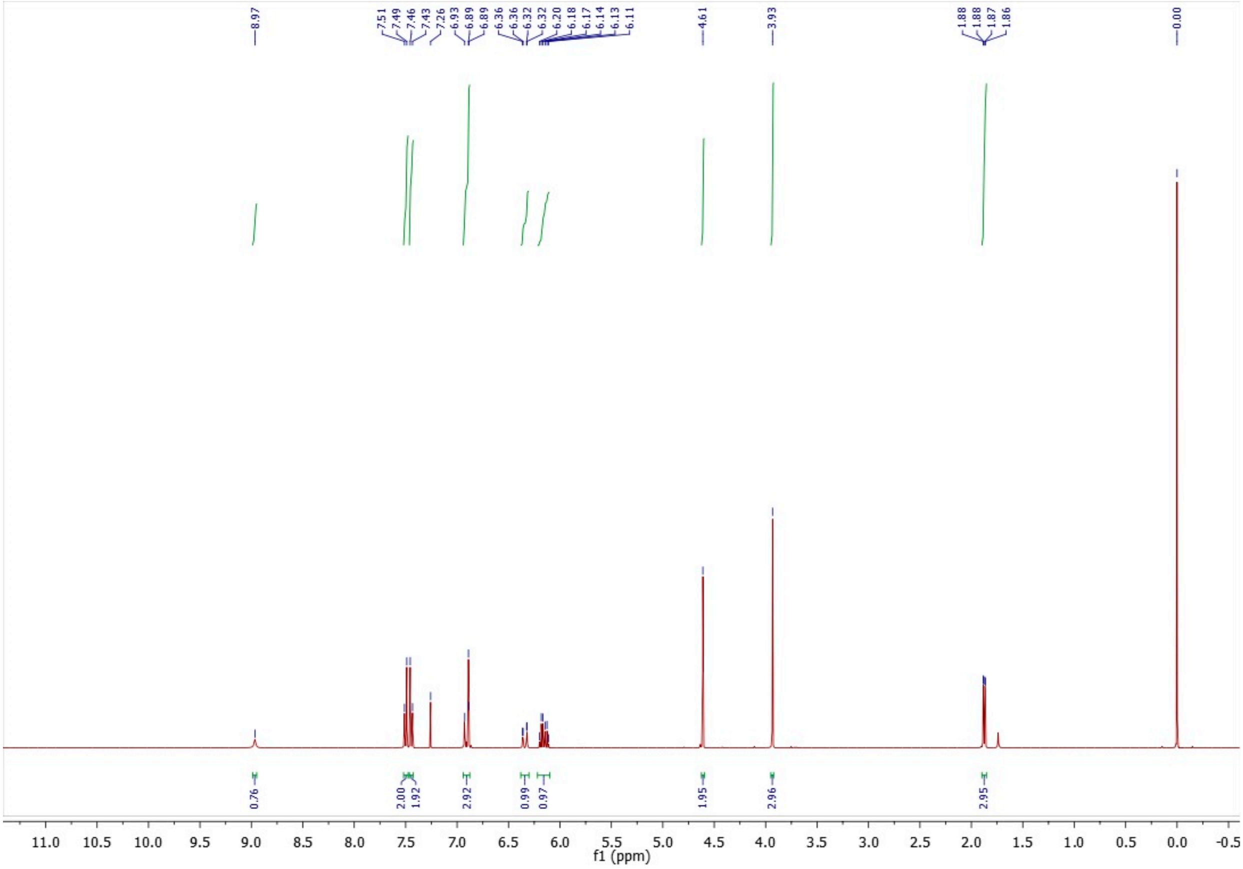

Figure S13: ^{1}H NMR spectrum (400 MHz, $CDCl_3$) of compound ISOA5.

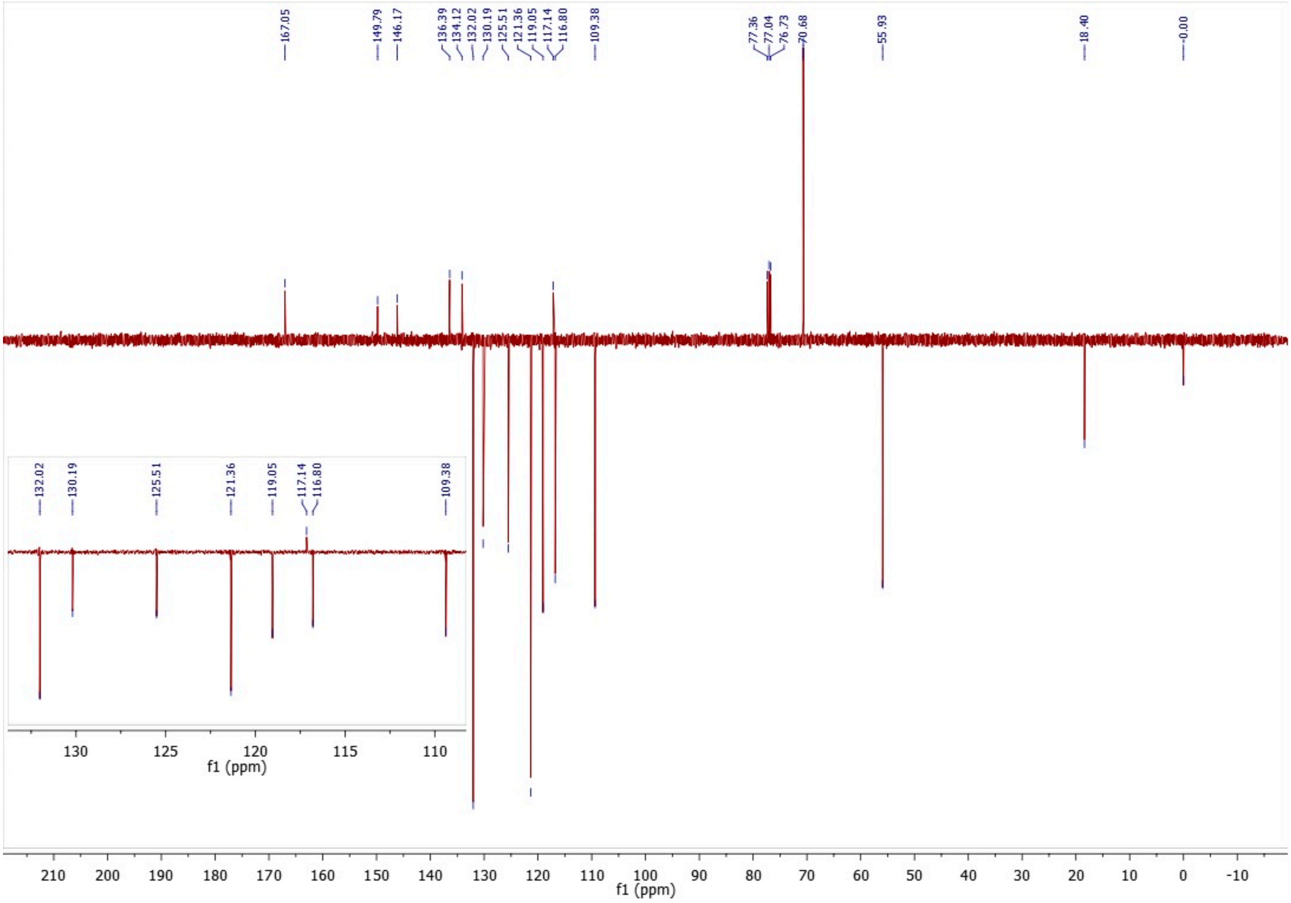

Figure S14: ^{13}C NMR spectrum (101 MHz, $CDCl_3$) of compound ISOA5.

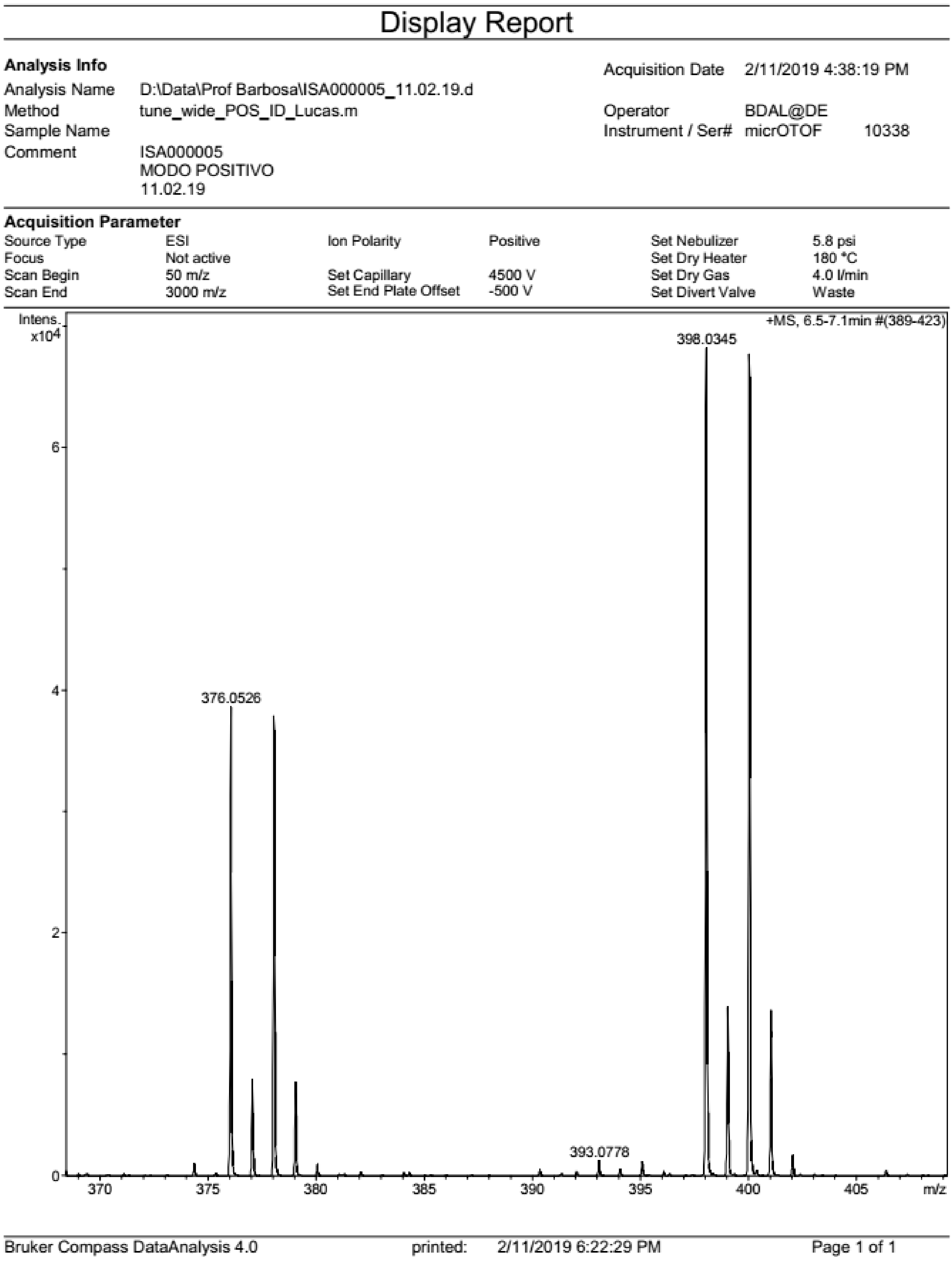

Figure S15: Mass spectrum of compound ISOA5.

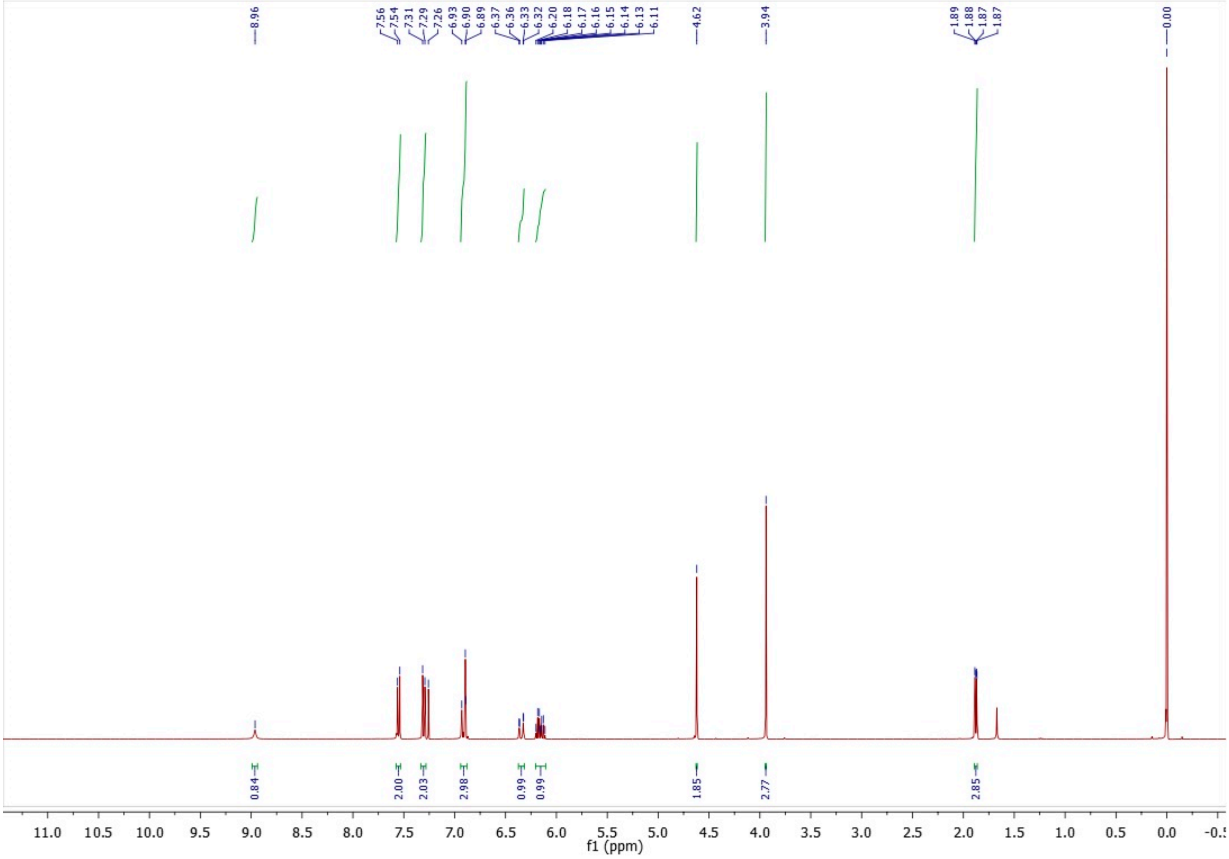

Figure S16: ^{1}H NMR spectrum (400 MHz, $CDCl_3$) of compound ISOA6.

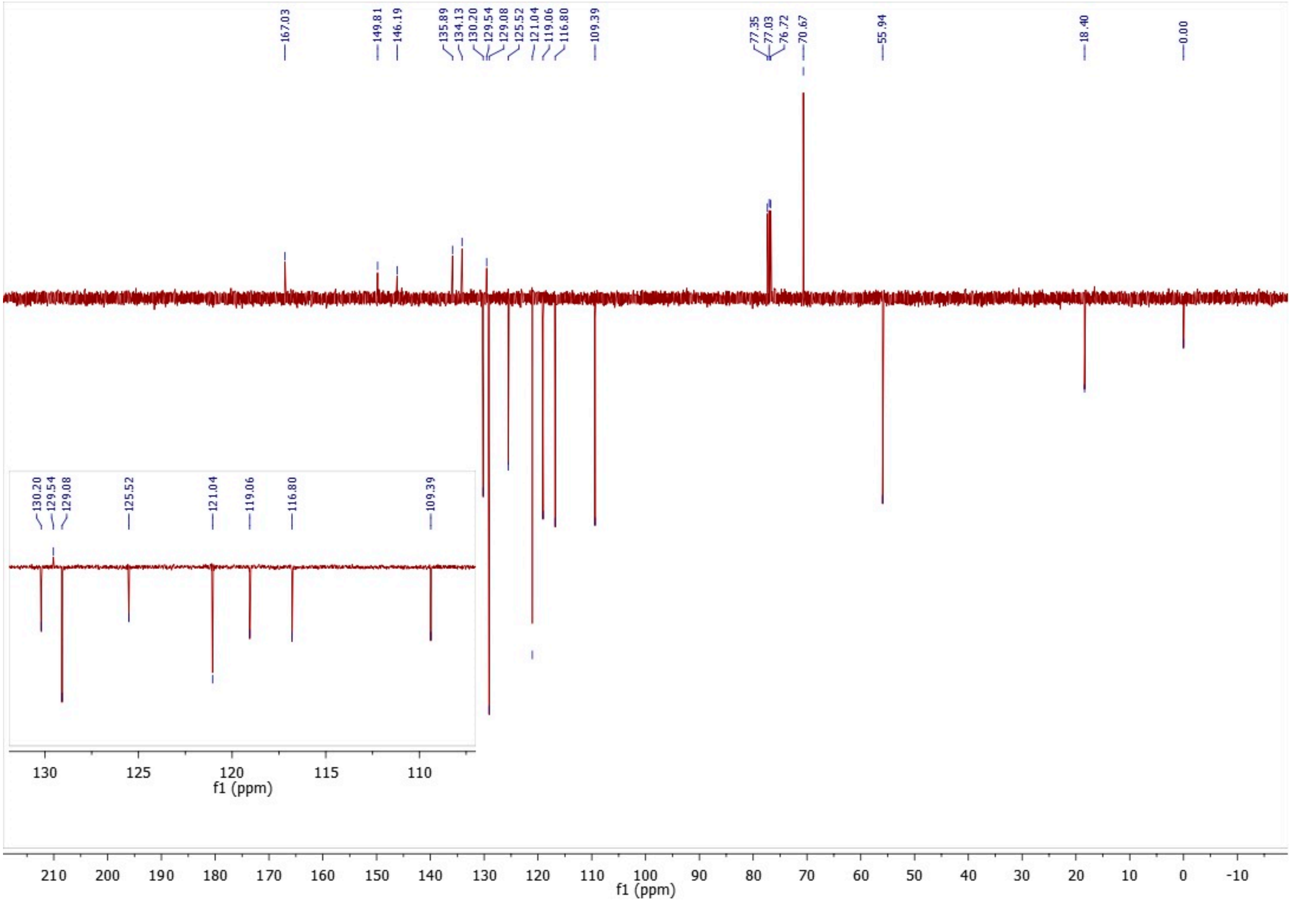

Figure S17: ^{13}C NMR spectrum (101 MHz, $CDCl_3$) of compound ISOA6.

Display Report

Analysis Info

Analysis Name	D:\Data\Prof Barbosa\ISA000006_11.02.19.d	Acquisition Date	2/11/2019 4:53:26 PM
Method	tune_wide_POS_ID_Lucas.m	Operator	BDAL@DE
Sample Name		Instrument / Ser#	micrOTOF 10338
Comment	ISA000006<br>MODO POSITIVO<br>11.02.19		

Acquisition Parameter

Source Type	ESI	Ion Polarity	Positive	Set Nebulizer	5.8 psi
Focus	Not active			Set Dry Heater	180 °C
Scan Begin	50 m/z	Set Capillary	4500 V	Set Dry Gas	4.0 l/min
Scan End	3000 m/z	Set End Plate Offset	-500 V	Set Divert Valve	Waste

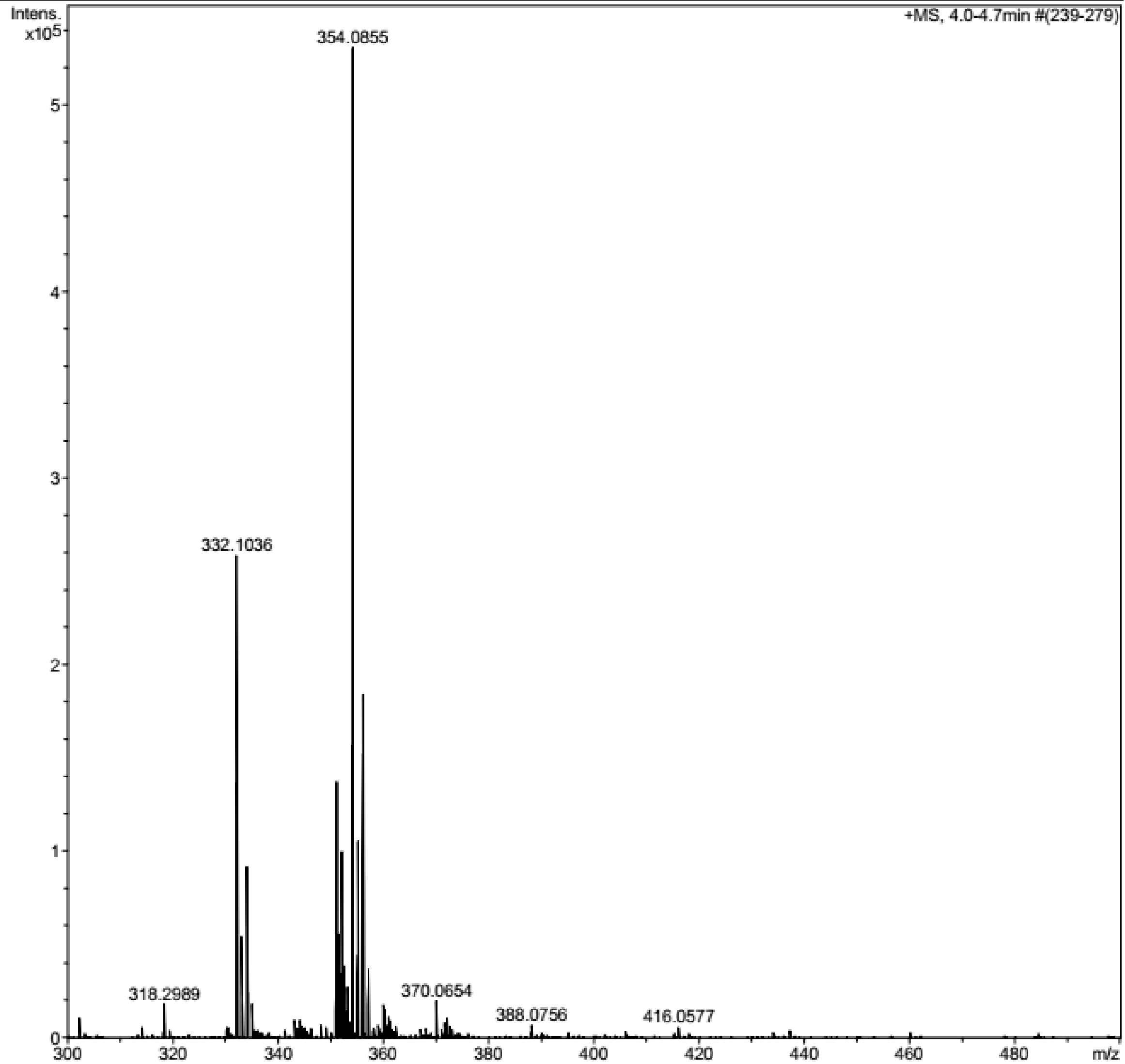

Bruker Compass DataAnalysis 4.0 printed: 2/11/2019 6:22:05 PM Page 1 of 1

Figure S18: Mass spectrum of compound ISOA6.

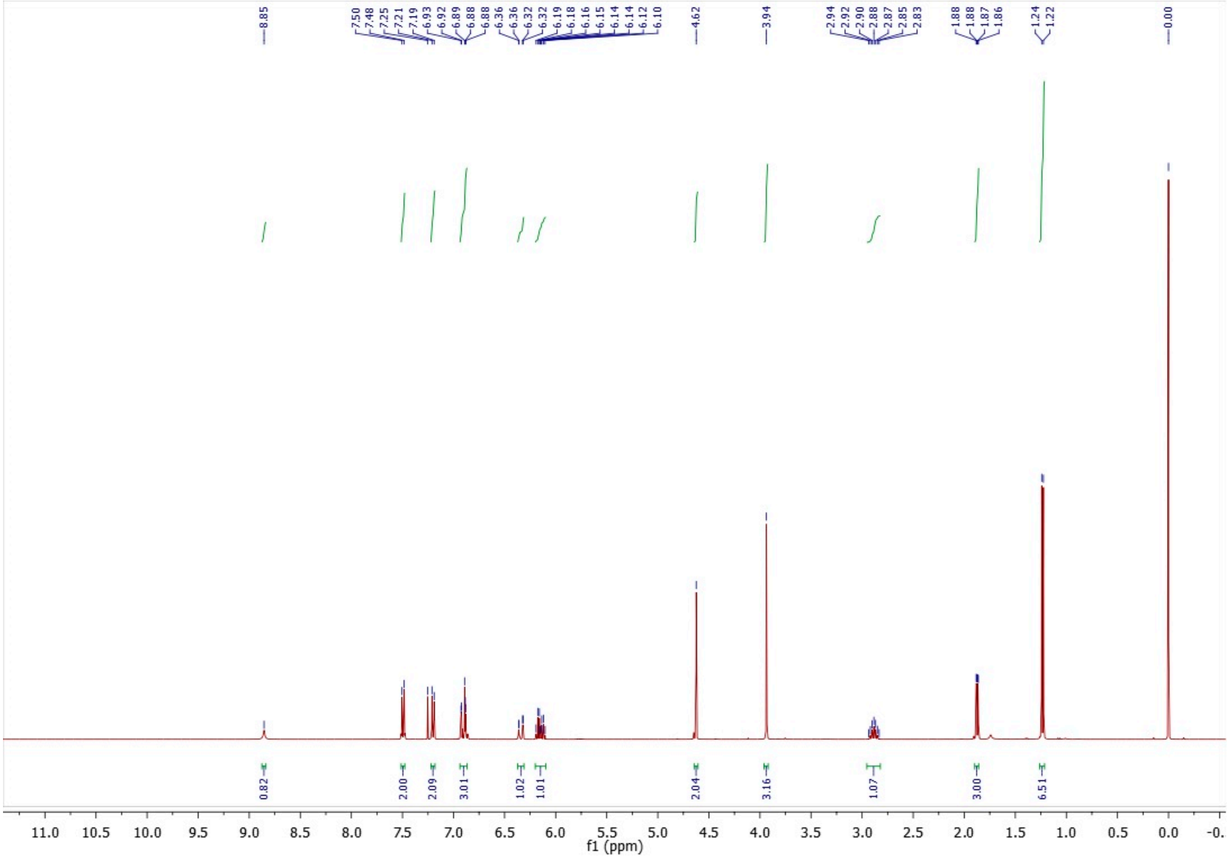

Figure S19: ^{1}H NMR spectrum (400 MHz, $CDCl_3$) of compound ISOA7.

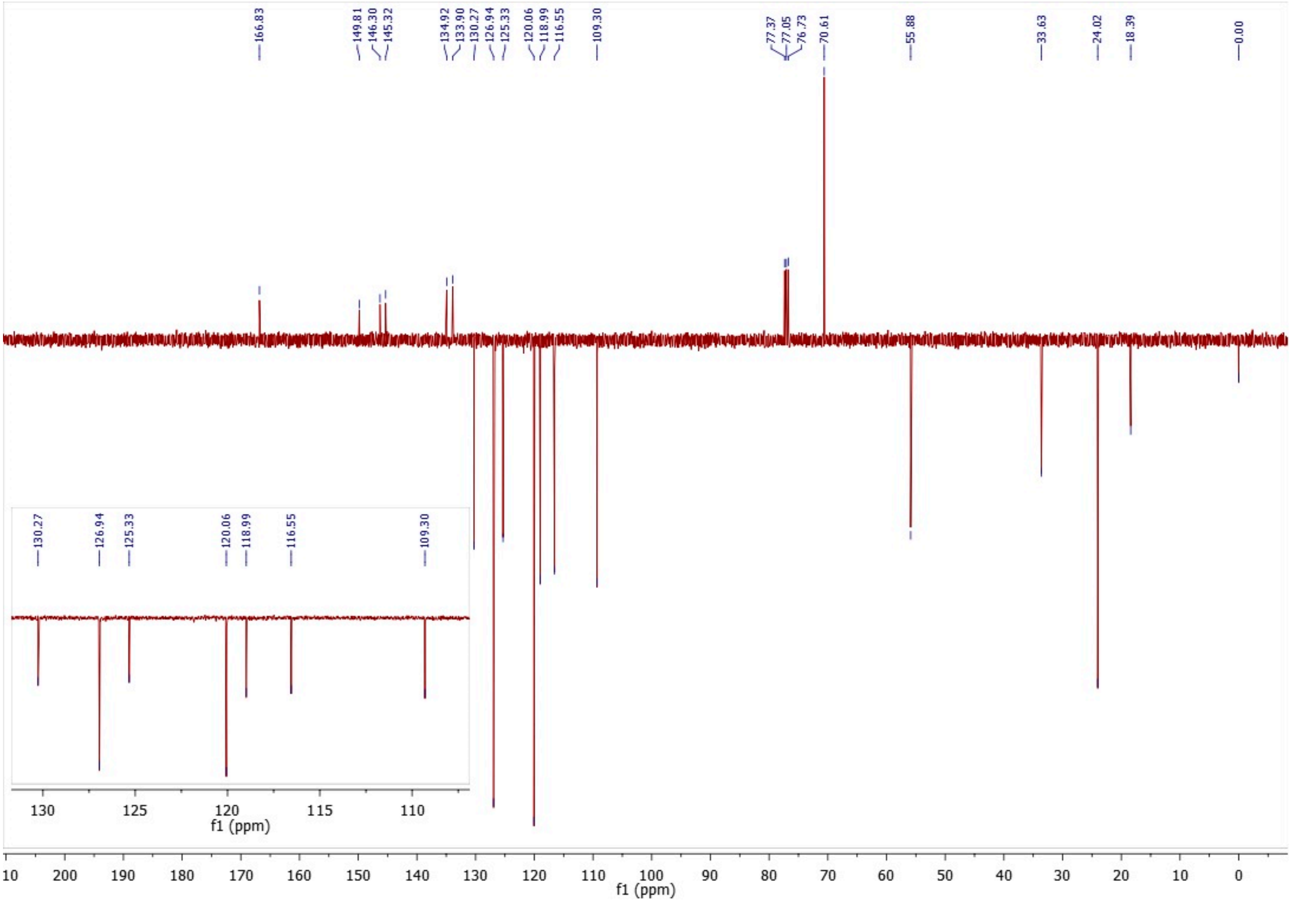

Figure S20: ^{13}C NMR spectrum (101 MHz, $CDCl_3$) of compound ISOA7.

Display Report

Analysis Info

Analysis Name	D:\Data\Prof Barbosa\ISA000007_11.02.19.d	Acquisition Date	2/11/2019 5:03:31 PM
Method	tune_wide_POS_ID_Lucas.m	Operator	BDAL@DE
Sample Name		Instrument / Ser#	micrOTOF 10338
Comment	ISA000007<br>MODO POSITIVO<br>11.02.19		

Acquisition Parameter

Source Type	ESI	Ion Polarity	Positive	Set Nebulizer	5.8 psi
Focus	Not active			Set Dry Heater	180 °C
Scan Begin	50 m/z	Set Capillary	4500 V	Set Dry Gas	4.0 l/min
Scan End	3000 m/z	Set End Plate Offset	-500 V	Set Divert Valve	Waste

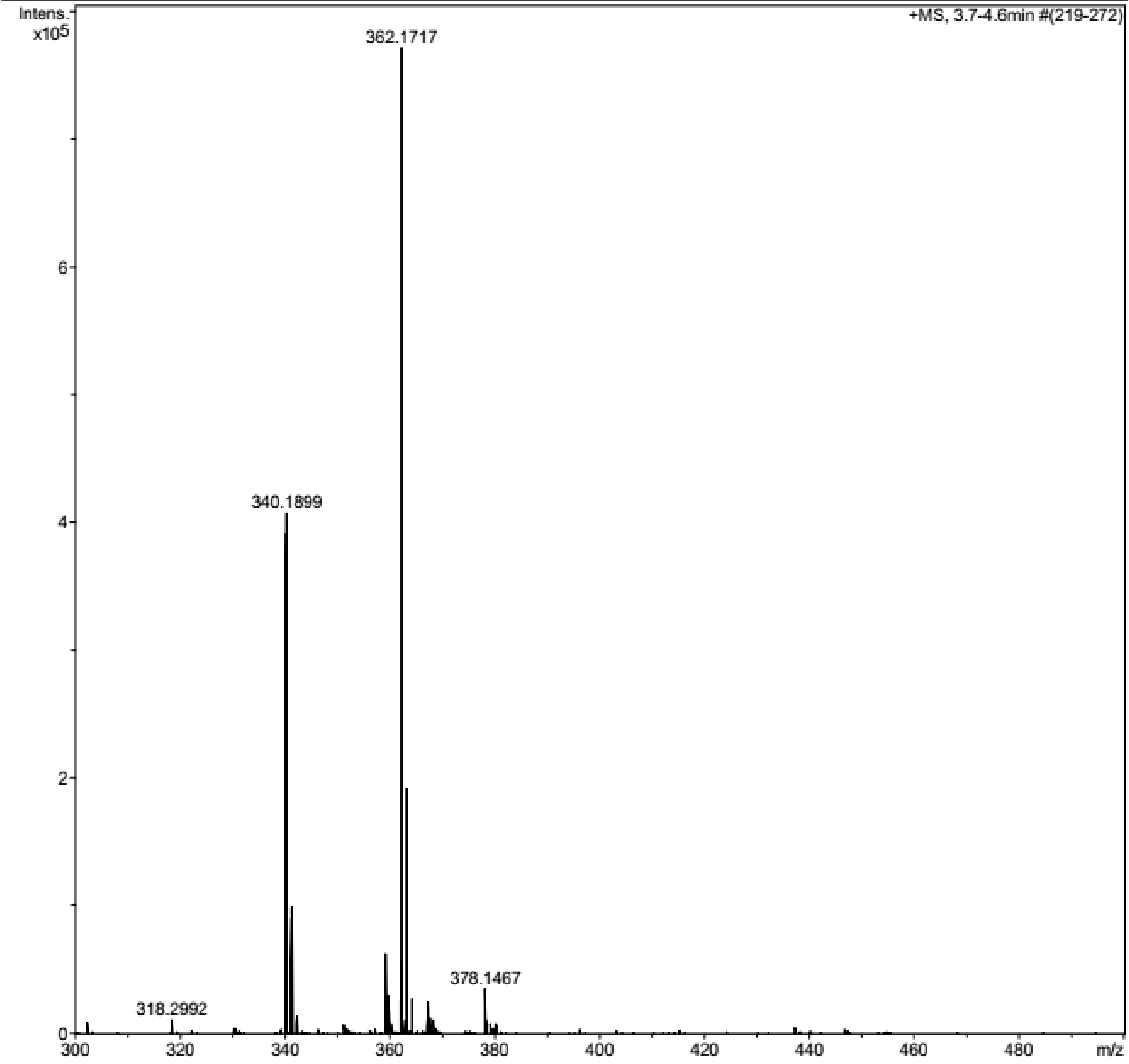

Bruker Compass DataAnalysis 4.0 printed: 2/11/2019 6:20:46 PM Page 1 of 1

Figure S21: Mass spectrum of compound ISOA7.

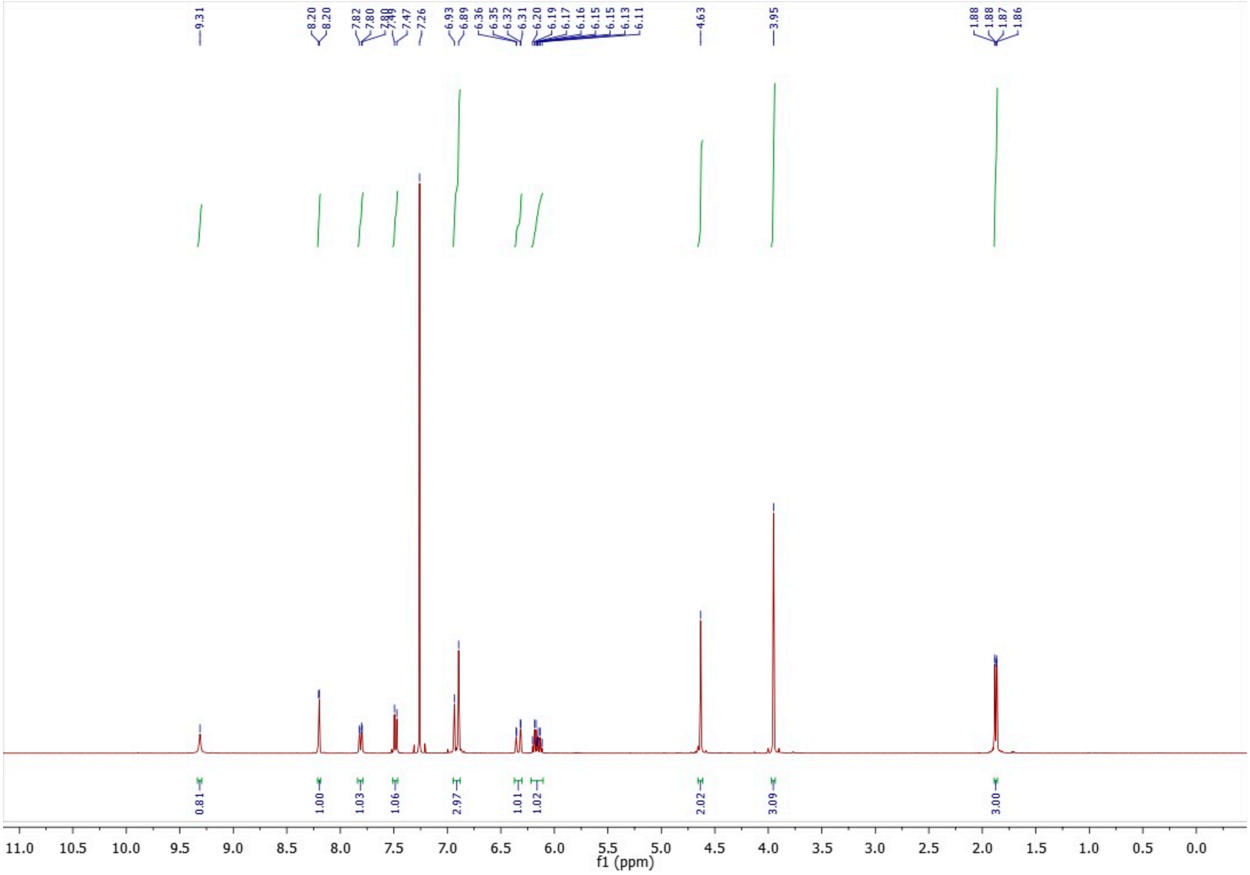

Figure S22: ^{1}H NMR spectrum (400 MHz, $CDCl_3$) of compound ISOA10.

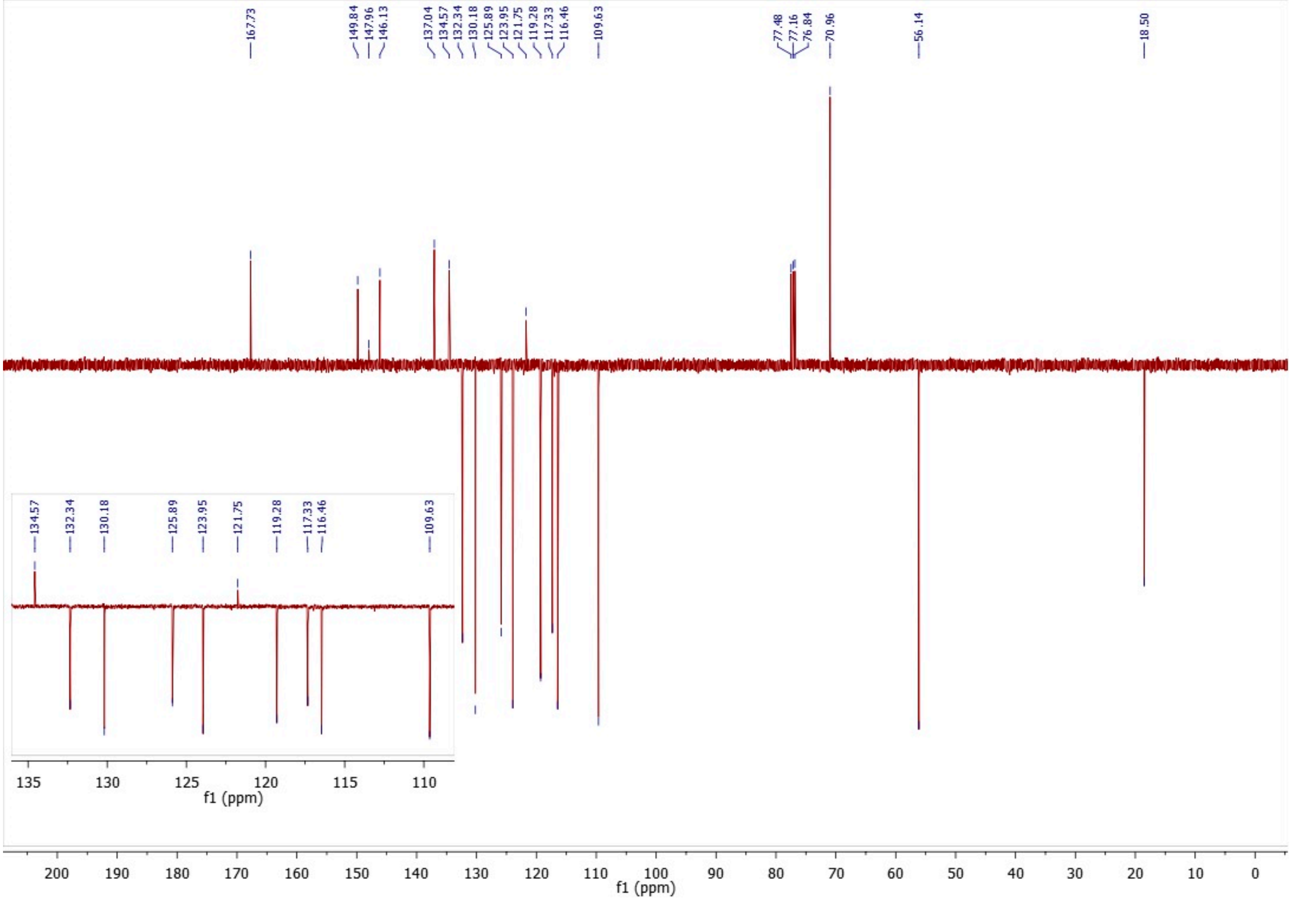

Figure S23: ^{13}C NMR spectrum (101 MHz, $CDCl_3$) of compound ISOA10.

Display Report

Analysis Info

Analysis Name	D:\Data\joanda\G_ISOA10_4_pos_1-8_01_641.d	Acquisition Date	17/3/2020 21:28:31
Method	tune_low_POS_LC_15MIN_tunemix_ok_psi_40.6_hex_150.m	Operator	BDAL@DE
Sample Name	G_ISOA10_4_pos	Instrument / Ser#	micrOTOF 10338
Comment			

Acquisition Parameter

Source Type	ESI	Ion Polarity	Positive	Set Nebulizer	40.6 psi
Focus	Not active			Set Dry Heater	300 °C
Scan Begin	50 m/z	Set Capillary	4500 V	Set Dry Gas	8.5 l/min
Scan End	3000 m/z	Set End Plate Offset	-500 V	Set Divert Valve	Waste

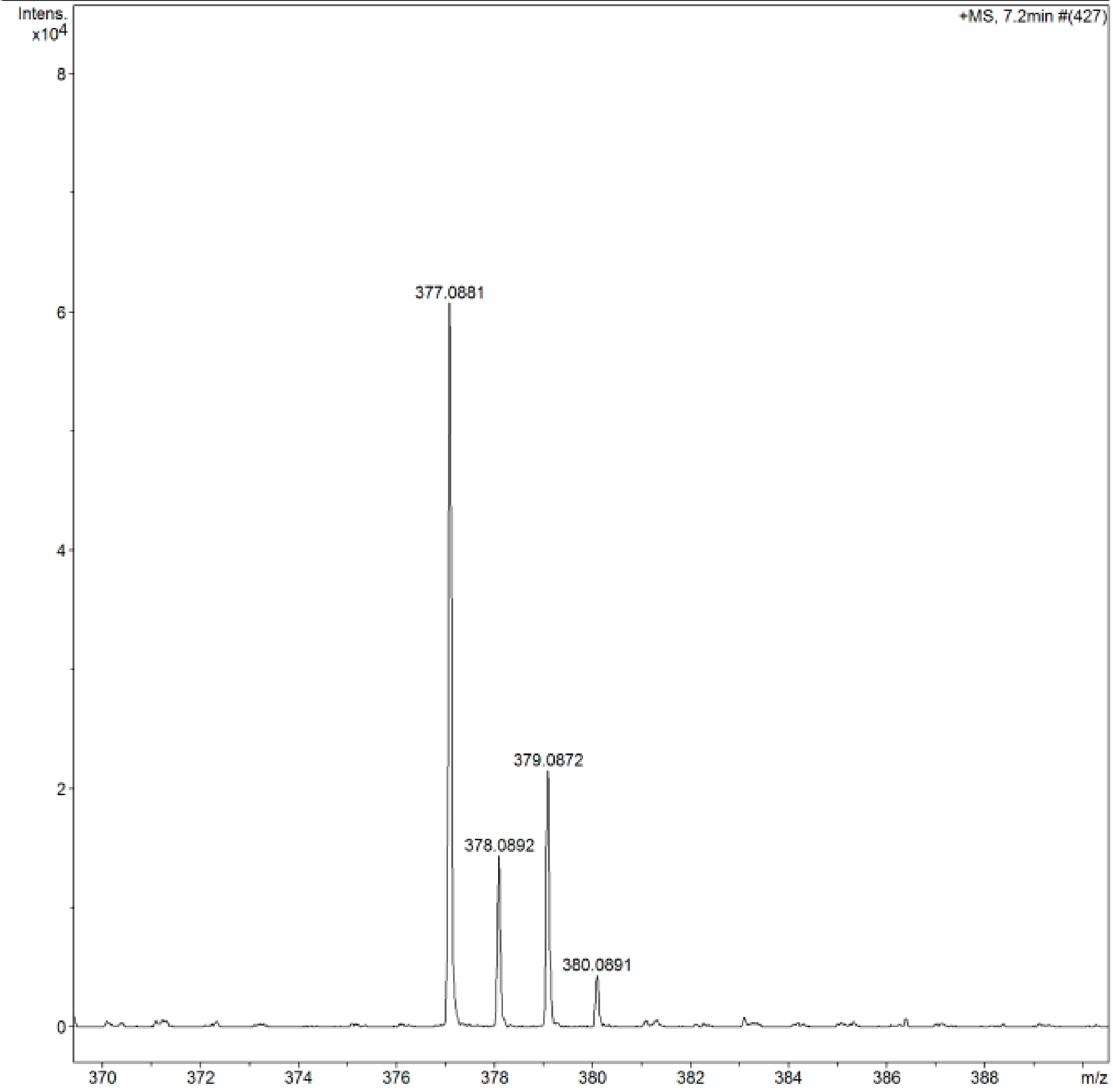

Bruker Compass DataAnalysis 4.0 printed: 17/3/2020 22:35:41 Page 1 of 1

Figure S24: Mass spectrum of compound ISOA10.

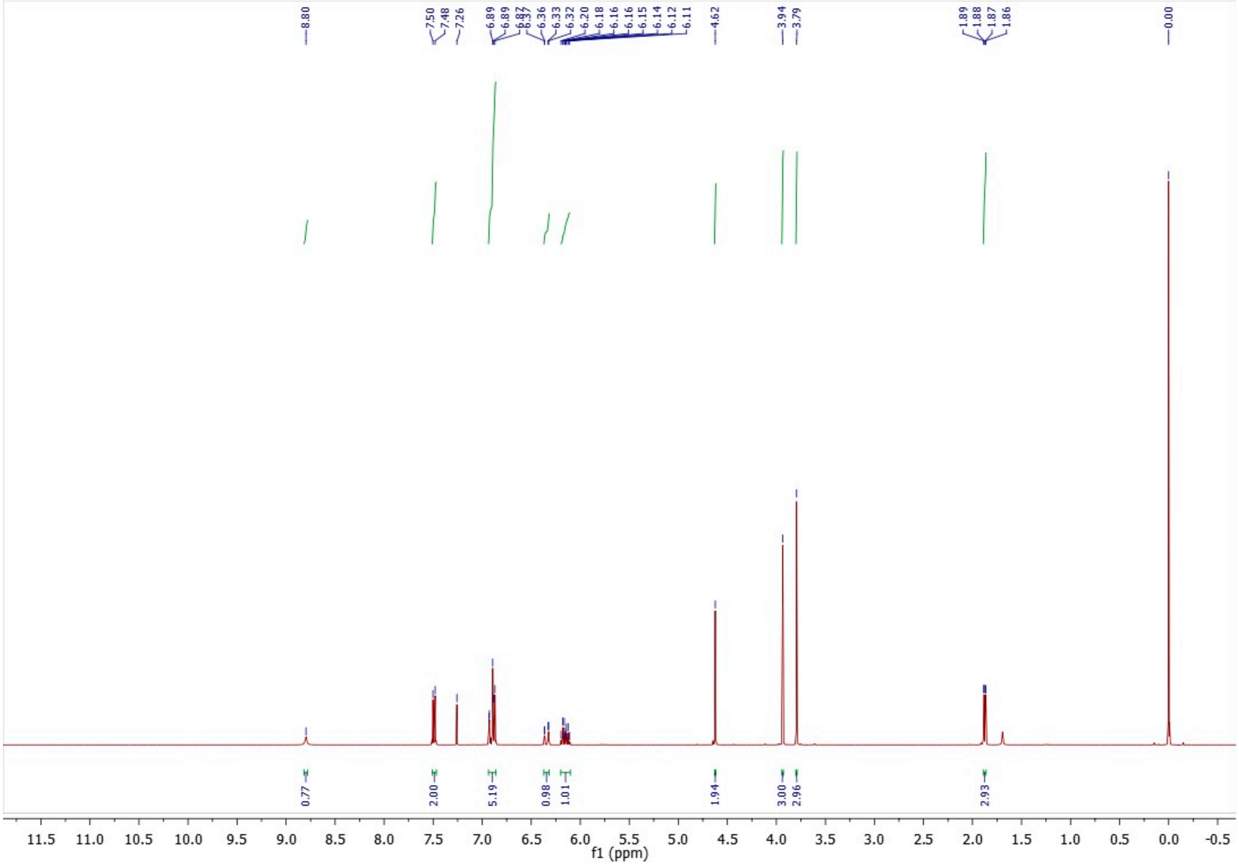

Figure S25: ^{1}H NMR spectrum (400 MHz, $CDCl_3$) of compound ISOA11.

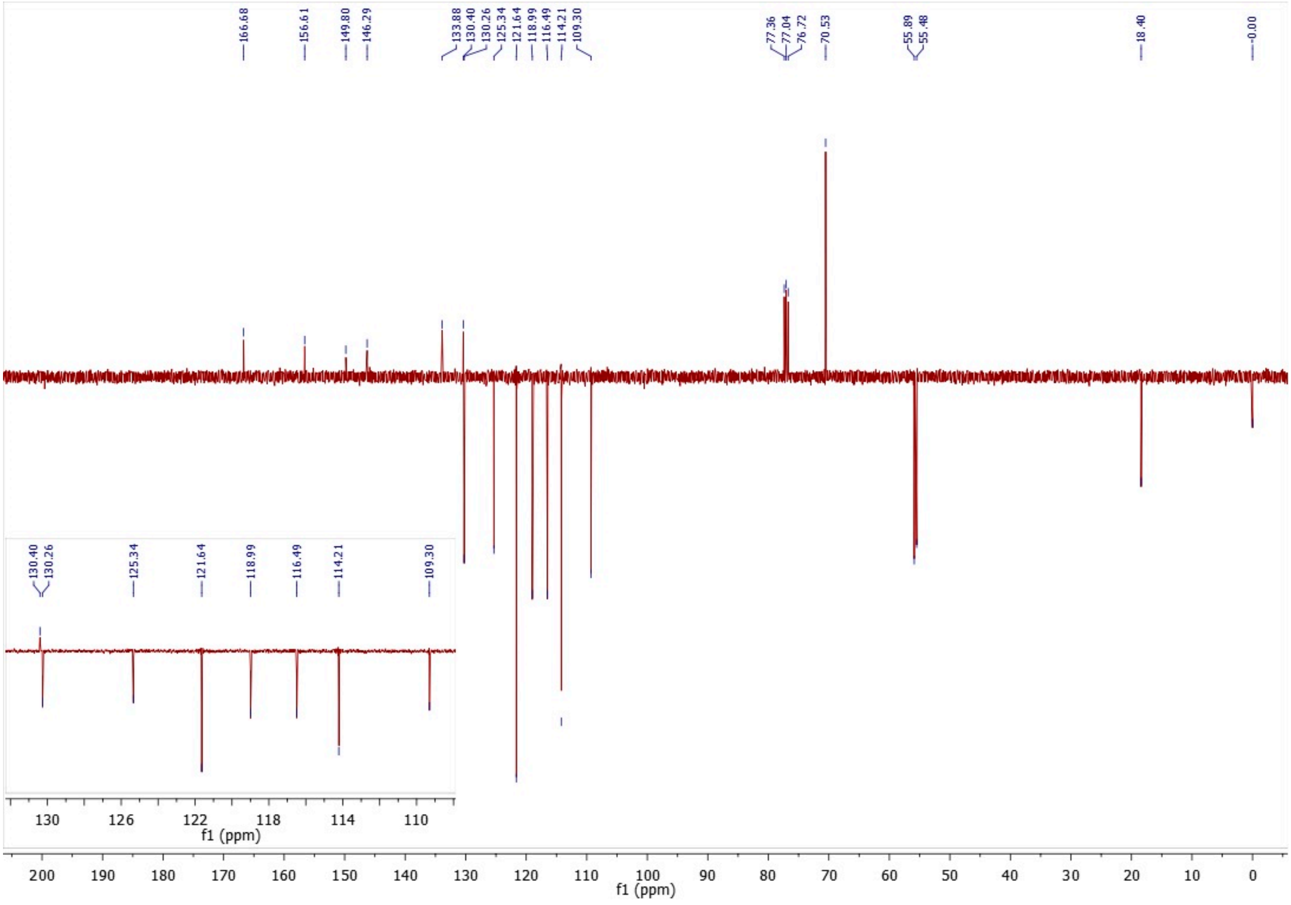

Figure S26: ^{13}C NMR spectrum (101 MHz, $CDCl_3$) of compound ISOA11.

Display Report

Analysis Info

Analysis Name	D:\Data\Prof Barbosa\ISA000011.02_11.02.19.d	Acquisition Date	2/11/2019 5:25:42 PM
Method	tune_wide_POS_ID_Lucas.m	Operator	BDAL@DE
Sample Name		Instrument / Ser#	micrOTOF 10338
Comment	ISA000011.02<br>MODO POSITIVO<br>11.02.19		

Acquisition Parameter

Source Type	ESI	Ion Polarity	Positive	Set Nebulizer	5.8 psi
Focus	Not active			Set Dry Heater	180 °C
Scan Begin	50 m/z	Set Capillary	4500 V	Set Dry Gas	4.0 l/min
Scan End	3000 m/z	Set End Plate Offset	-500 V	Set Divert Valve	Waste

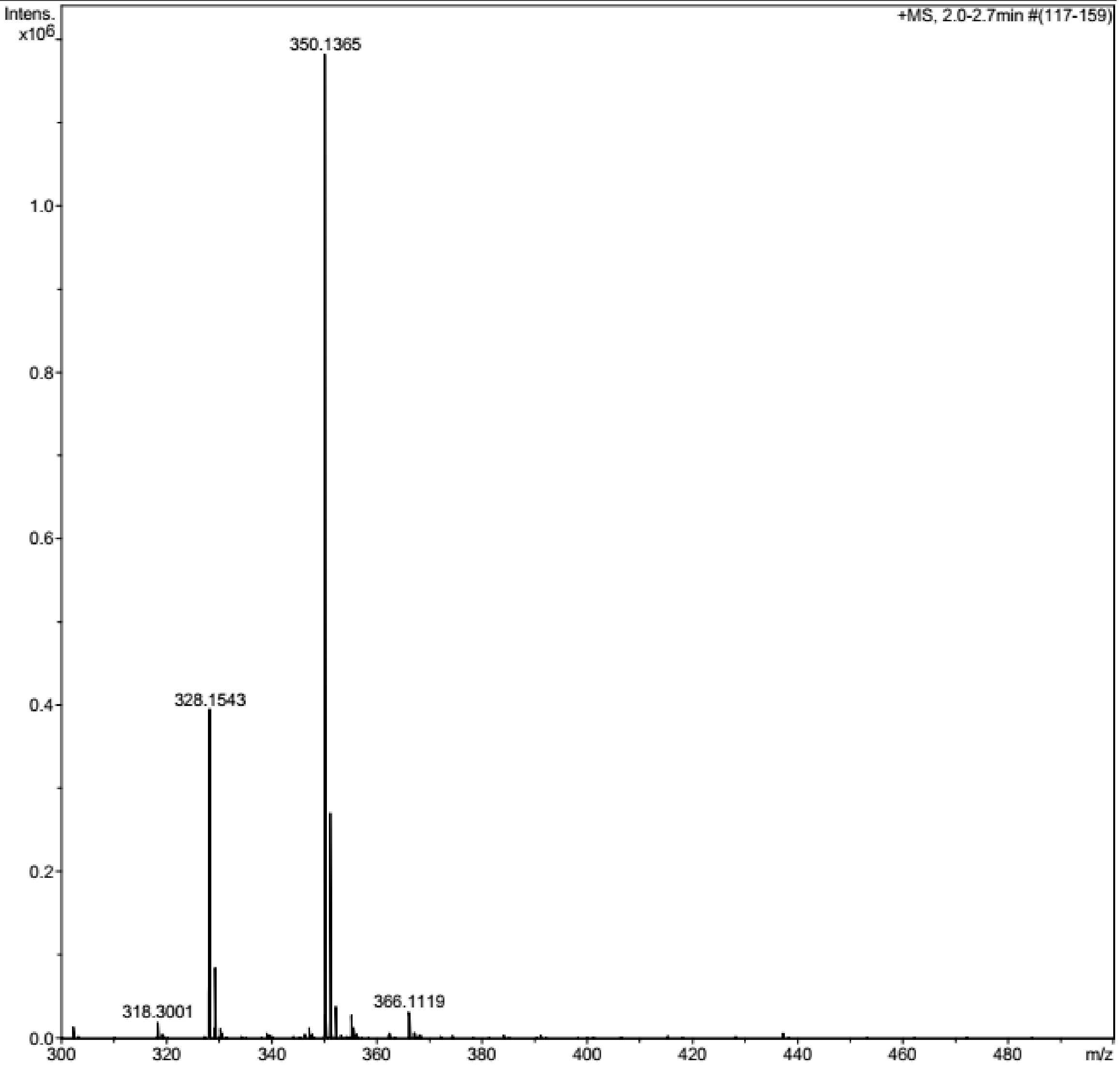

Bruker Compass DataAnalysis 4.0 printed: 2/11/2019 6:20:19 PM Page 1 of 1

Figure S27: Mass spectrum of compound ISOA11.

Rerefences

1. Luis, J. A. S.; Souza, H. D. S.; Lira, B. F.; Alves, F. S.; Athayde-Filho, P. F.; Lima, T. K. S.; Rocha, J. C.; Junior, F. J. B. M.; Scotti, L.; Scotti, M. T. Combined structure- and ligand-based virtual screening aiding discovery of selenoglycolicamides as potential multitarget agents against *Leishmania* species. *J Mol Struct* **2019**, *1189*, 126872.

© 2020 by the authors. Submitted for possible open access publication under the terms and conditions of the Creative Commons Attribution (CC BY) license (http://creativecommons.org/licenses/by/4.0/).
